# CCT complex restricts neuropathogenic protein aggregation via autophagy

**DOI:** 10.1038/ncomms13821

**Published:** 2016-12-08

**Authors:** Mariana Pavel, Sara Imarisio, Fiona M. Menzies, Maria Jimenez-Sanchez, Farah H. Siddiqi, Xiaoting Wu, Maurizio Renna, Cahir J. O'Kane, Damian C. Crowther, David C. Rubinsztein

**Affiliations:** 1 Department of Medical Genetics, University of Cambridge, Cambridge Institute for Medical Research, Wellcome Trust/MRC Building, Addenbrooke's Hospital, Hills Road, Cambridge CB2 2XY, UK; 2 Department of Genetics, University of Cambridge, Downing Street, Cambridge CB2 3EH, UK; 3 MedImmune Limited, Aaron Klug Building, Granta Park, Cambridge CB21 6GH, UK

## Abstract

Aberrant protein aggregation is controlled by various chaperones, including CCT (chaperonin containing TCP-1)/TCP-1/TRiC. Mutated CCT4/5 subunits cause sensory neuropathy and CCT5 expression is decreased in Alzheimer's disease. Here, we show that CCT integrity is essential for autophagosome degradation in cells or *Drosophila* and this phenomenon is orchestrated by the actin cytoskeleton. When autophagic flux is reduced by compromise of individual CCT subunits, various disease-relevant autophagy substrates accumulate and aggregate. The aggregation of proteins like mutant huntingtin, ATXN3 or p62 after CCT2/5/7 depletion is predominantly autophagy dependent, and does not further increase with CCT knockdown in autophagy-defective cells/organisms, implying surprisingly that the effect of loss-of-CCT activity on mutant ATXN3 or huntingtin oligomerization/aggregation is primarily a consequence of autophagy inhibition rather than loss of physiological anti-aggregation activity for these proteins. Thus, our findings reveal an essential partnership between two key components of the proteostasis network and implicate autophagy defects in diseases with compromised CCT complex activity.

## Abstract (web summary)

The CCT complex, a key player in the chaperone machinery, has been implicated in Huntington's disease. Pavel *et al*. show that CCT2/5/7 also play an essential role in autophagosome degradation, and that the aggregation of proteins upon CCT2/5/7 depletion is primarily a consequence of impaired autophagy.

Intracellular protein misfolding and aggregation are seen in many late-onset neurodegenerative diseases, called proteinopathies, such as Alzheimer's disease, Parkinson's disease, tauopathies, and the nine polyglutamine expansion diseases, exemplified by Huntington's disease. In the autosomal dominant forms of these conditions, most of the mutations confer novel toxic functions on the specific protein. Thus, the phenotypic severity in model systems frequently correlates with the levels of the relevant proteins^1^. The accumulation and aggregation of such proteins is buffered by the proteostasis network that regulates either the concentrations or folding of intracellular clients. The concentration of such intracytoplasmic clients is regulated in part by degradation pathways, including the ubiquitin–proteasome pathway and (macro)autophagy. Autophagy is a lysosomal degradation pathway where damaged cellular material and long-lived proteins are engulfed into double-membraned structures named autophagosomes, which ultimately fuse with lysosomes where their contents are degraded. Since mutant huntingtin, p62, tau and many other intracytoplasmic neurodegenerative disease-associated aggregate-prone proteins are autophagy substrates, the levels of these proteins increase in both the soluble and aggregated states when autophagy is compromised^1^,^2^,^3^.

Protein folding is assisted by the chaperone machinery. Here, one key player is chaperonin containing TCP-1 (CCT, also known as TRiC or group II chaperonin), a cytosolic ATP-dependent eukaryotic chaperonin comprising two rings of eight different but related subunits, each thought to be represented once per eight-membered ring. Autosomal recessive mutations of the *CCT5* and *CCT4* subunits lead to loss-of function phenotypes that manifest with a devastating sensory neuropathy^4^,^5^. Moreover, recent studies suggested that mRNA levels of the TRiC complex are repressed in Alzheimer's disease patient brain samples^6^. This might be important, as tau, which accumulates in Alzheimer's disease, is a client protein of many chaperones and co-chaperones (Hsp90/CHIP and Hsp70 complexes), including CCT, that together control both its stabilization and degradation^7^. Therefore it is important to understand the mechanistic consequences of impaired CCT activity in neurodegenerative diseases. TRiC was initially thought to fold only the cytoskeletal proteins actin and tubulin but is now known to handle a wide range of cytoplasmic clients^8^,^9^.

A role for CCT as a folding enhancer has been suggested by studies showing that it can prevent mutant huntingtin (htt) aggregation by direct binding to the aggregate-prone N-terminal of htt *in vitro*^10^,^11^,^12^. Recent studies also showed that the apical domain of CCT1 subunit is able to decrease the formation of visible inclusions and oligomeric mutant htt-exon1 fragment species independently of the TRIC complex *per se*^13^. A recent study observed that both CCT3 and the apical domain of CCT1 (ApiCCT1) reduced htt levels in cortical neurons from an Huntington's disease mouse model, which was associated with normalized anterograde BDNF transport, restored retrograde BDNF transport and normalized lysosomal transport^14^. Accordingly, we and others anticipated that the main role of this chaperone was to directly regulate the aggregation of huntingtin and related substrates. While this direct binding/sequestration mechanism represents an intuitive mechanism for CCT to regulate mutant huntingtin aggregation, indirect mechanisms dependent on degradation pathways have not been tested, or excluded previously. Here we report our unexpected findings that loss of CCT function increases the accumulation of well characterized aggregate-prone proteins, like the N-terminal mutant htt fragment, mainly as a consequence of autophagy inhibition.

## Results

### CCT is required for autophagosome degradation

**Figure 1:**
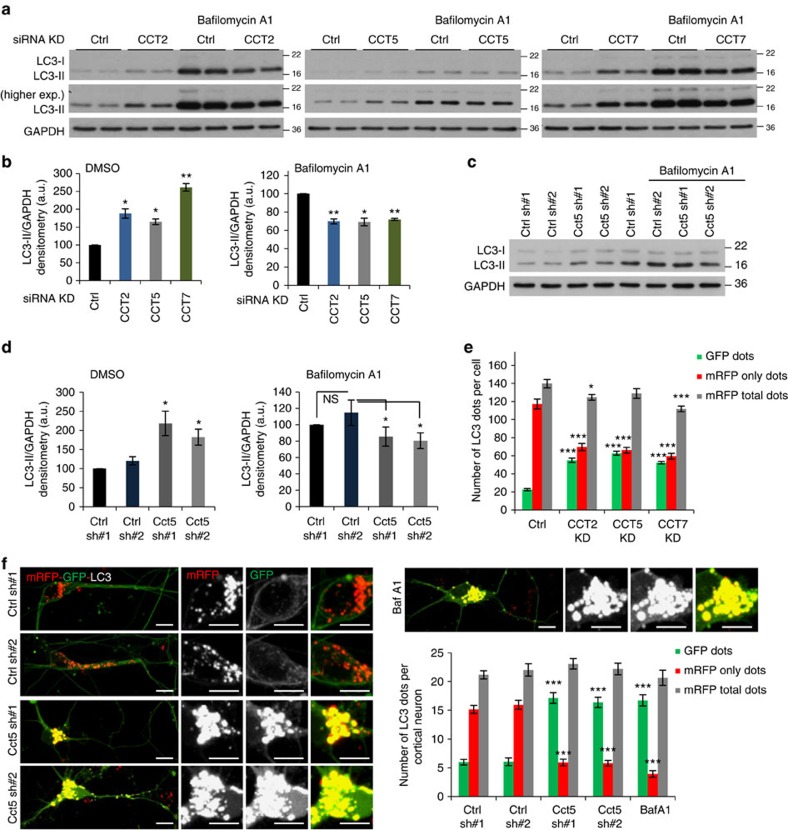
CCT knockdown reduces LC3 turnover and autophagosome formation. (**a**) LC3-II levels in HeLa cells transfected with siRNA against scrambled control or CCT subunits treated with vehicle (DMSO) or Bafilomycin A1 (400 nM) for 5 h. GAPDH is loading control. (**b**) LC3-II/GAPDH densitometry from **a**. Graphs show means±s.d. of triplicate samples in the absence or presence of Bafilomycin A1. Similar results were observed in at least three independent experiments. (*n*=3; **P<0.01, *P<0.05; two-tailed one sample *t*-test). For combined data of independent biological replicates, see Supplementary Fig. 1c. (**c**) LC3-II levels in mouse primary cortical neurons transduced with two different shRNA lentiviral particles targeting either control (Ctrl sh#1 is empty vector and Ctrl sh#2 is control scramble) or the mouse Cct5 (Cct5 sh#1 and Cct5 sh#2). Cells were treated with vehicle (DMSO) or Bafilomycin A1 at 200 nM for 12 h. GAPDH is loading control. (**d**) LC3-II/GAPDH densitometry from **c**. Graphs show mean±s.d. of triplicate samples in the absence or presence of Bafilomycin A1. Similar results were observed in another two independent experiments. In Bafilomycin A1 conditions, the Cct5 shRNAs (#1 or #2) treated cells showed significant reduced LC3-II levels when compared with the scramble control (Ctrl sh#2) treated neurons (*n*=3; *P<0.05, NS, not significant; two-tailed *t*-test), but not-significant if compared with the empty-vector control (Ctrl sh#1) condition (*n*=3; NS, not significant; two-tailed one sample *t*-test). (**e**) GFP dot numbers (autophagosomes/non-acidified autolysosomes, mRFP-GFP double labelled vesicles) and mRFP-only dots (acidified autolysosomes) in HeLa cells. HeLa cells stably expressing mRFP-GFP-LC3 were treated as in **a** then seeded in a 96-well plate and subjected to automated Cellomics visualization and analysis. Graph shows results from one representative experiment. We analysed 100–200 cells per well (from a 96-well plate) with 10–12 wells per condition. Similar data were seen in other two independent experiments. Bars—means±s.e.m. (***P<0.001; two-tailed *t*-test). (**f**) Representative confocal z-stack images (left panel) and total number of GFP dots (autophagosomes) and mRFP-only dots (autolysosomes)—left panel, in mouse primary cortical neurons from mRFP-GFP-LC3 transgenic mice. Cortical neurons were plated in live cell dishes, treated as in **d** and the numbers of dots were manually counted after confocal microscopy. Graph shows data from three independent biological replicates; 100–150 neurons counted per each condition. Bars—means±s.e.m. (***P<0.001; two-tailed *t*-test). Scale bar—10 μm.

To test if the chaperonin complex played a role in autophagy, we designed either siRNA knockdown or lentiviral shRNA transduction experiments targeting individual subunits of the complex (CCT2/5/7)—all eight subunits are required for the proper function of the chaperonin complex and depletion of any of them will reduce its activity^10^. As this is a complex, knockdown of one subunit may impact the levels of other components—(indeed, we observed that CCT2 knockdown also reduces CCT7 subunit levels, see Supplementary Figs 1a and 13 for full scans of blots in the paper). Autophagy was quantified by measuring the levels of LC3-II, a classical marker of autophagosome abundance^15^. LC3-II levels on western blots might vary between control and treatment conditions as a result of changes in the formation and/or degradation of autophagosomes^16^,^17^. To distinguish between these two possibilities, we exposed the cells to saturating concentrations of the lysosomal V-ATP-ase inhibitor Bafilomycin A1 (BafA1), which blocks LC3-II/ autophagosome degradation^18^,^19^ and reveals changes in autophagosome synthesis. Depletion of individual CCT subunits in HeLa cells or mouse primary cortical neurons (depletion efficiency in Supplementary Fig. 1a,b) increased the steady state levels of LC3-II in the absence of Bafilomycin A1. In the presence of Bafilomycin A, these knockdowns resulted in a modest consistent LC3-II decrease in HeLa cells (Fig. 1a,b; Supplementary Fig. 1c) and neurons (when we compare the targeting to scramble shRNAs; Fig. 1c,d). The large increase in autophagosome number and modest decrease in autophagosome formation (LC3-II in the presence of BafA1) suggest that depletion of individual CCT subunits primarily impairs degradation of autophagosomes in both cell types, and this also appears to be associated with a modest decrease in autophagosome biogenesis. We confirmed the block in autophagosome degradation with another assay based on measuring the autophagy flux in HeLa cells and primary cortical neurons of mice transgenically expressing the tandem tagged mRFP-GFP-LC3 reporter^20^,^21^ (Supplementary Fig. 1d). This assay allows one to characterize the LC3-II vesicles and discriminate between autophagosomes or non-acidified lysosomes, and acidified autolysosomes. Due to the different *p*Ka of the two LC3 tags (<4.5 for mRFP and around 6 for GFP), the GFP signal is rapidly quenched by the acidic lysosomal environment, while the mRFP persists^20^,^21^. Thus, the vesicles that emit both red and green signals are autophagosomes or non-acidified lysosomes, while the vesicles that emit only the red signal (due to the GFP-signal being quenched by an acidic environment) are acidic autolysosomes. Knockdowns of CCT complex subunits (CCT2/5/7) increased the number and size of autophagosomes/non-acidified lysosomes (yellow vesicles/GFP dots), while the number of acidic autolysosomes (red only vesicles/mRFP only dots) was significantly decreased (Fig. 1e,f; Supplementary Fig. 1e,f). These data were consistent with our previous western blot based results showing a block in LC3-II degradation/reduced autophagy flux. For these experiments, BafA1 was used as a positive control for impaired autophagosome flux/degradation.

### CCT regulates lysosomal functioning

The previous results showing a block in autophagosome degradation under CCT depletion conditions may be due to decreased lysosomal activity/degradation (reduced autophagosome degradation), impaired autophagosome maturation with reduced autophagosome-lysosome fusion, or both. To clarify which of these situations caused the block in autophagosome degradation in CCT-depleted cells, we tested each hypothesis (impaired autophagosome-lysosome fusion or decreased lysosomal acidification) by performing appropriate assays.

**Figure 2:**
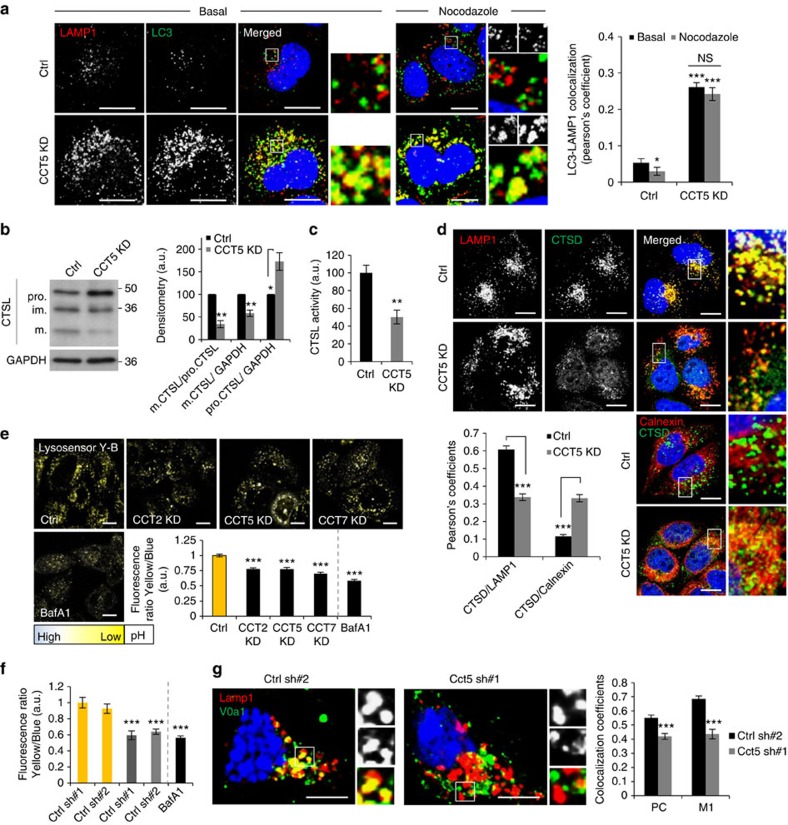
CCT is required for lysosomal functioning and autophagosomes degradation. (**a**) LC3 (anti-mouse Alexa 488) and LAMP1 (anti-rabbit Alexa 568) colocalization in HeLa cells depleted for CCT5. Right panel: quantification of colocalization (Pearson's coefficient). More than 30 cells were counted per condition. Bars—means±s.e.m. (***P<0.001, *P<0.05, NS, not significant; two-tailed *t*-test). Similar results were achieved in at least two independent experiments. Scale bar, 10 μm. (**b**) Immunoblot showing Cathepsin L (CTSL) maturation in CCT5 knockdown HeLa cells. Graph showing the quantification of mature/precursor CTSL ratio is on right as mean±s.d. (*n*=3; **P<0.01, *P<0.05; two-tailed one sample *t*-test). (**c**) Cathepsin L activity in HeLa cells depleted for CCT5. Fluorescence intensity was expressed as percentage of control. Bars—mean±s.d. (*n*=3; **P<0.01; two-tailed *t*-test). (**d**) Cathepsin D (CTSD, anti-mouse Alexa 488)—LAMP1 (anti-rabbit Alexa 568) and cathepsin D (anti-mouse Alexa 488) —calnexin (anti-rabbit Alexa 568) double-immunostaining in CCT5-knockdown cells. More than 40 cells were quantified per experiment (each experiment was performed at least twice with similar results). Bars—mean of Pearson's coefficient±s.e.m. (***P<0.001; two-tailed *t*-test). Scale bar—10 μm. See also Supplementary Fig. 6e. (**e**) Lysosomal pH in CCT-depleted cells. The Lysosensor Yellow Blue intensity was measured in both yellow and blue channels. Intensities ratio is a qualitative measure of lysosomal pH. Bafilomycin A1 (Baf A1) was used as a positive control (400 nM, 3 h) for increased lysosomal pH >50 cells were analysed per experiment, for each condition. Experiment was performed at least 3 times with similar results. Bars—mean±s.e.m. (***P<0.001; two-tailed *t*-test). (**f**) Lysosomal pH in Cct5-depleted mouse primary cortical neurons. Lysosensor Yellow Blue intensity was measured as in **e**. Bafilomycin A1 was used as a positive control (400 nM, 6 h) for increased lysosomal pH >40 neurons were analysed per experiment and per condition. Experiment was performed at least three times with similar results. Bars—means±s.e.m. (***P<0.001; two-tailed *t*-test). See also Supplementary Fig. 6f. (**g**) Voa1 (anti-rabbit Alexa555) and Lamp1 (anti-rat Alexa647) double-immunostaining in mouse primary cortical neurons. Neurons were plated and treated as in **f**. More than 30 neurons were counted per condition. PC is Pearson's coefficient; M1 is Mander's coefficient 1, which indicates the Voa1 localization in the Lamp1 compartment. Bars—means±s.e.m. (***P<0.001; two-tailed *t*-test). Scale bar—10 μm. See also Supplementary Fig. 6g.

To assess autophagosome-lysosome fusion in control or CCT-depleted cells, we initially quantified and characterized LC3 (autophagosome marker) and LAMP1/CD63 (lysosome/late endosome markers) double-labelled vesicles. Knockdown of individual chaperonin subunits (CCT2/5/7) increased the colocalization of endogenous LC3 with LAMP1, suggesting that autophagosome-lysosome fusion was not impaired in HeLa cells (Fig. 2a,b; Supplementary Fig. 2a,b) and the same phenomenon was seen when we knocked down Cct5 in primary mouse cortical neurons (Supplementary Fig. 2c,d). This was not an artefact due to increased autophagosome size/number or clustering, as the same phenomenon (increased LC3/LAMP1 colocalization compared with control) was seen after we briefly exposed cells to the microtubule-depolymerizing drug nocodazole which disperses lysosomes uniformly in the cell and dissociates them from the non-fused autophagosomes found in their close proximity^22^ (Fig. 2a; Supplementary Fig. 2a–b). Indeed, nocodazole treatment significantly reduced LC3-LAMP1 colocalization by at least 20–30% in control cells (as previously reported^22^), but had no such effect in CCT2/5/7 knockdown cells, when compared with cells without nocodazole (Fig. 2a; Supplementary Fig. 2a,b). These data were reinforced by double immunostaining for endogenous p62 (an autophagic cargo) and CD63 (another late endosome/lysosome marker) in ordinary HeLa cells or HeLa cells stably expressing GFP-LC3 exposed to CCT5 siRNAs. The colocalizations of endogenous p62/CD63 (Supplementary Fig. 3), GFP-LC3/endogenous CD63 (Supplementary Fig. 4) and endogenous p62/GFP-LC3 (Supplementary Fig. 4) were increased upon CCT5 knockdown compared with control cells. Note that Bafilomycin A1, used as a control in these experiments, likely affects both autophagosome–lysosome fusion as well as autophagosome degradation^23^.

To understand the functionality of the lysosomes, we initially observed that CCT depletion caused the enlargement of LAMP1/CD63-positive compartments and their redistribution from a preferentially perinuclear localization in control cells to a scattered phenotype in CCT-deficient cells, phenomena commonly associated with compromised lysosomal function (Fig. 2a; Supplementary Figs 2a,3a,4a). At this point, it is important to mention that many LAMP1-positive vesicles may be amphisomes or autophagosome-nonacidic autolysosome fusions, as LAMP1/CD63 showed significantly increased colocalization with LC3, in CCT2/5/7 knockdown HeLa cells or Cct5-depleted primary cortical neurons, or with p62, in CCT5 knockdown HeLa cells (Fig. 2a; Supplementary Figs 2–4). Lysosomal function relies on the capacity of lysosomal hydrolytic enzymes to become activated in an acidic environment of about pH 4.5. Indeed, the maturation and *in vitro* activity of the lysosomal protease cathepsin L (CTSL) was decreased by depletion of individual CCT subunits in HeLa (Fig. 2b,c; Supplementary Fig. 5a,b) and mouse primary cortical neurons (Supplementary Fig. 5c,d). Reduced maturation of cathepsins can result from intracellular mistrafficking and/or increased lysosomal pH (for example, see effects with BafA1 in Supplementary Fig. 5)^24^. Consistent with intracellular mistrafficking of lysosomal enzymes, we observed increased reticular staining for cathepsin D (CTSD) and less localization in the lysosomal compartment in CCT-depleted cells (Fig. 2d; Supplementary Fig. 6a,b). This was associated with increased colocalization between cathepsin D and calnexin, an ER marker (Fig. 2d; Supplementary Fig. 6c–e), indicating mistrafficking of lysosomal enzymes and their sequestration in the ER. We next measured lysosomal pH with the ratiometric- and pH-sensitive probe LysoSensor Yellow/Blue by live-cell imaging. The LysoSensor Yellow/Blue provides the advantage of simultaneous dual-emission, predominantly yellow fluorescence in an acidic environment and blue in neutral and less-acidic vesicles. The information provided by this probe is based on the ratio of yellow to blue intensity and therefore is independent of potential differences in probe uptake between treated and control cells. CCT subunit knockdowns elevated the lysosomal pH in HeLa cells (Fig. 2e) with a more dramatic phenotype in primary cortical neurons, where Cct5 was depleted by lentiviral transduction (reduced yellow/blue ratio, BafA1 was used as a positive control) (Fig. 2f; Supplementary Fig. 6f). The enhanced lysosomal pH (here observed in CCT-depleted cells) also correlates with the enlargement and redistribution of lysosomes towards cell periphery^25^,^26^. This pH disturbance is likely due to reduced delivery of the Voa1 (a subunit of the V-ATPase proton pump) to lysosomes, which indeed showed less localization in the LAMP1 compartment in Cct5-depleted primary mouse cortical neurons (Fig. 2g; Supplementary Fig. 6g). These data (reduced lysosomal acidification and uncompromised autophagosome–lysosome fusion) are consistent with previous observations that V-ATPase defects affect only autophagosome degradation *per se* and not their fusion with lysosomes^27^. Additionally, this phenotype of non-acidified lysosomes associated with apparently normal autophagosome–lysosome fusion is seen in ATP13A2 deficiency^28^. These results suggest that the autophagosome degradation impairment seen in CCT-depleted cells is a direct consequence of reduced lysosomal acidification which is likely due to impaired V-ATPase trafficking.

**Figure 3:**
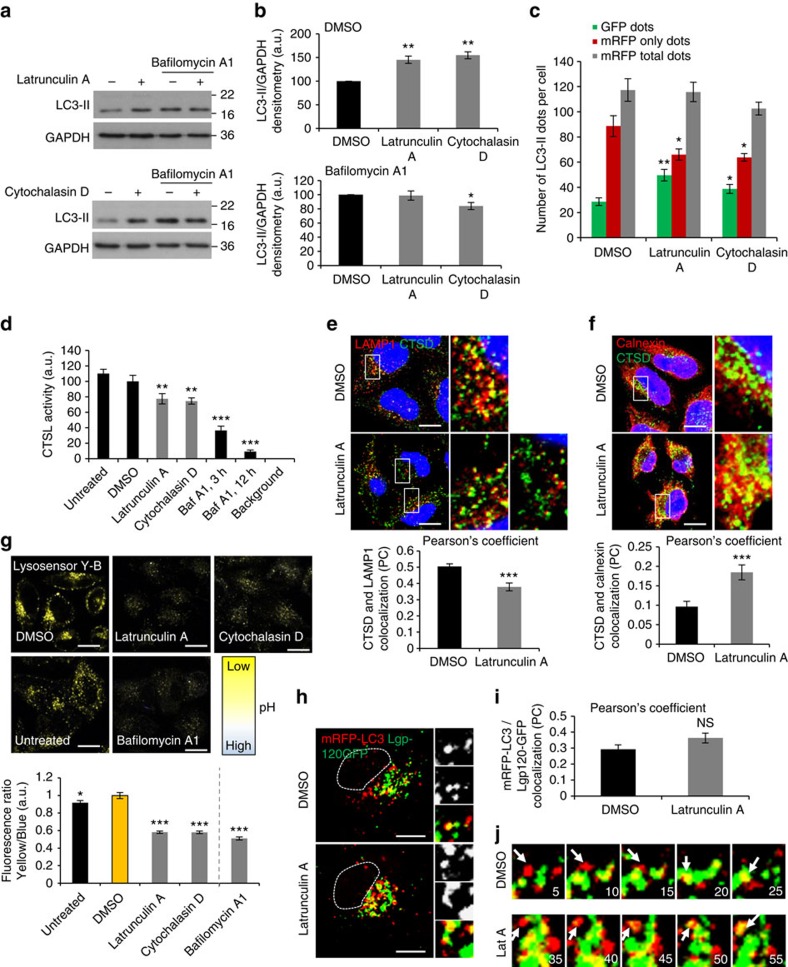
Depolymerization of actin cytoskeleton reduces the lysosomal biogenesis. (**a**) LC3-II levels in HeLa cells treated with latrunculin A (1 μM) or cytochalasin D (1 μM) for 3 h in the presence or absence of Bafilomycin A1 (400 nM, 3 h). (**b**) LC3-II/GAPDH densitometry in HeLa cells treated with Latrunculin A or Cytochalasin D as in **a**. Bars—means±s.d. (*n*=3; **P<0.01; *P<0.05; two-tailed one sample *t*-test). (**c**) GFP dot numbers (autophagosomes) and mRFP-only dots (autolysosomes) in HeLa cells stably expressing mRFP-GFP-LC3 and independently treated with 1 μM latrunculin A or cytochalasin D for 3 h. Graphs show the results for one representative experiment: eight wells of 100–200 cells each were analysed per sample. Experiment was repeated with similar results. Bars—means±s.e.m. (*n*=8; **P<0.01, *P<0.05; two-tailed *t*-test) (**d**) Cathepsin L (CTSL) activity in HeLa cells treated with the indicated drugs (1 μM, 3 h). Bafilomycin was positive control (400 nM, 3 h). Bars—means±s.d. (*n*=3; ***P<0.001, **P<0.01; two-tailed *t*-test). (**e**) Cathepsin D (CTSD, anti-mouse Alexa 488) and LAMP1 (anti-rabbit Alexa 568) double-immunostaining in cells treated with latrunculin A (1 μM, 3 h). Bottom graph—colocalization quantification. More than 30 cells were counted per experiment (each experiment was performed at least twice). Bars—mean±s.e.m. (***P<0.001; two-tailed *t*-test). Scale bar, 10 μm. (**f**) Cathepsin D (anti-mouse Alexa 488) and calnexin (anti-rabbit Alexa 568) double-immunostaining in latrunculin A-treated cells (1 μM, 3 h). Bottom panel—colocalization quantification. More than 30 cells were counted per experiment (each experiment was performed at least twice). Bars—means±s.e.m. (***P<0.001; two-tailed *t*-test). Scale bar—10 μm. (**g**) Lysosomal pH in cells treated with actin disrupting drugs (latrunculin A, cytochalasin D; 3 h, 1 μM) in full medium. Bafilomycin A1—positive control for increased lysosomal pH. More than 50 cells were counted per experiment, performed twice. Bars—means±s.e.m., where DMSO control was set up to 1 (arbitrary units) (***P<0.001, *P<0.05; two-tailed *t*-test). (**h**–**j**) mRFP-LC3 and Lgp120-GFP live-cell imaging in HeLa cells exposed to Latrunculin A (1 μM). Autophagosome–lysosome fusion is not affected compared with the control. Scale bar, 10 μm. (**i**) mRFP-LC3 and Lgp120-GFP colocalization expressed as Pearson's coefficient. More than 25 cells were counted per experiment. Bars—means±s.e.m. (NS, not significant; two-tailed *t*-test). (**j**) Autophagosome–lysosome fusion events in HeLa cells expressing mRFP-LC3 and Lgp120-GF. For both control and Latrunculin A treatment, the movies show five frames compressed per second (from a total of 70 frames, taken continuously). Arrows—fusion events. Scale bar, 10 μm.

Actin is one of the major clients for CCT folding^29^,^30^ and, as expected, we observed that CCT knockdown disrupted the F-actin cytoskeleton (Supplementary Fig. 7a). Since actin depolymerization blocks autophagosome degradation^31^,^32^, we hypothesized that actin disruption mediated the lysosomal phenotypes caused by CCT depletion. If actin defects were responsible for the autophagy–lysosomal impairment seen in CCT-depleted conditions, then one would expect actin depolymerization to mirror the lysosomal dysfunction caused by CCT loss-of-function. Indeed, actin depolymerizing drugs (latrunculin A and cytochalasin D) impaired autophagosome clearance (Fig. 3a–c; Supplementary Fig. 7b–d), reduced cathepsin L maturation and activity (Fig. 3d; Supplementary Fig. 7e,f), decreased the amounts of cathepsin D in the lysosomal compartment (Fig. 3e; Supplementary Fig. 7g) with increased cathepsin D–calnexin colocalization (Fig. 3f; Supplementary Fig. 7h) and elevated lysosomal pH (Fig. 3g), all phenotypes seen with CCT subunit depletion. We next investigated autophagosome–lysosome fusion in cells transiently expressing both mRFP-LC3 (autophagosomes) and lgp120-GFP (LAMP1, lysosomes and late endosomes) exposed to short-term latrunculin A treatment. As predicted, autophagosome–lysosome fusion was not compromised under these circumstances (Fig. 3h–j).

**Figure 4:**
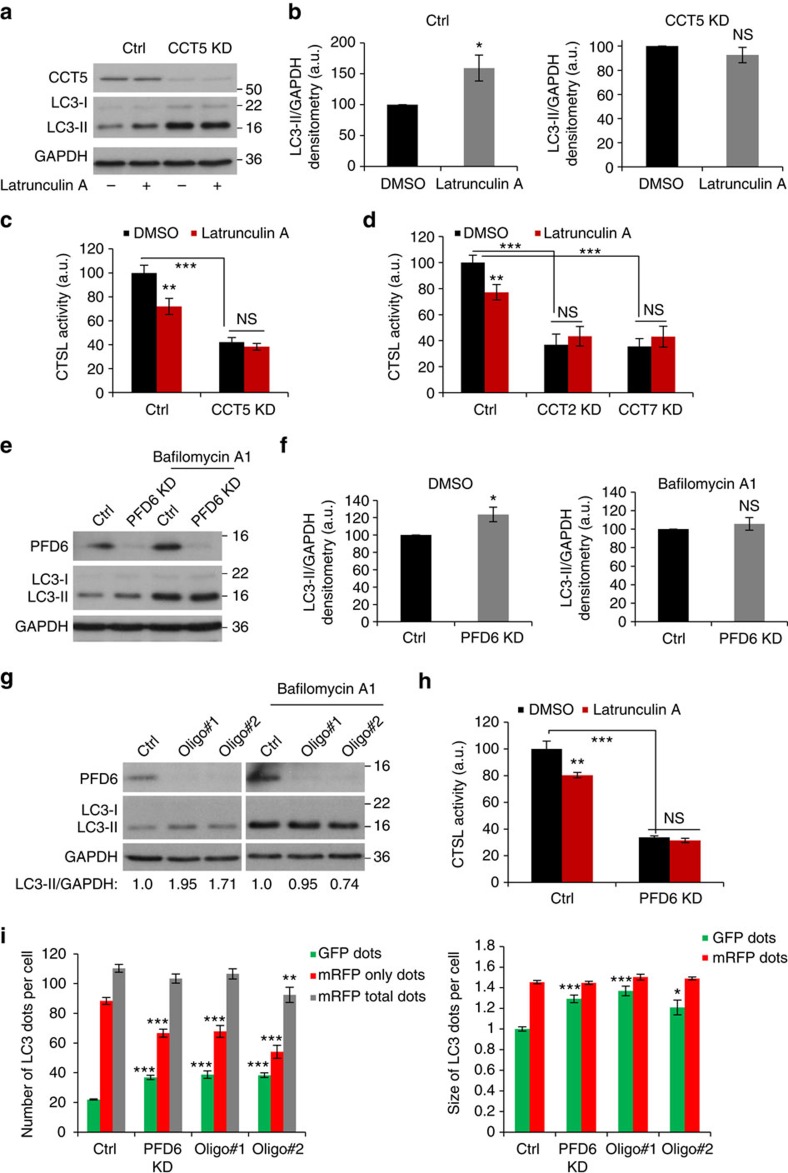
CCT controls lysosomes biogenesis via actin cytoskeleton. (**a**) LC3-II blots of HeLa cells treated with CCT5 siRNA and exposed to latrunculin A (1 μM, 3 h). (**b**) LC3-II/GAPDH densitometry in HeLa cells treated as in **a**. Bars—means±s.d. (*n*=3; *P<0.05, NS, not significant; two-tailed one sample *t*-test). (**c**) *In vitro* cathepsin L (CTSL) activity in HeLa cells exposed to CCT5 siRNA. Fluorescence intensity expressed as percentage of control. The cells were additionally exposed to Latrunculin A (3 h, 1 μM). Bars—means±s.d. (*n*=3; ***P<0.001, **P<0.01, NS, not significant; two-tailed *t*-test). (**d**) *In vitro* cathepsin L activity in HeLa cells exposed to CCT2 or CCT7 siRNAs. Fluorescence intensity was expressed as percentage of control. The cells were additionally exposed to Latrunculin A (3 h, 1 μM). Bars—means±s.d. (*n*=3; ***P<0.001, **P<0.01, NS, not significant; two-tailed *t*-test). (**e**) LC3-II levels in HeLa cells exposed to two rounds of PFD6 (prefoldin) smart-pool siRNA treatments in the presence/absence of Bafilomycin A1 (400 nM, 3 h). (**f**) LC3-II/GAPDH densitometry for HeLa cells exposed to two rounds of PFD6 (prefoldin) smart-pool siRNA and treated as in **e**. Bars—means±s.d. (*n*=3; *P<0.05, NS, not significant; two-tailed one sample *t*-test). (**g**) LC3-II blots of HeLa cells treated with individual PFD6 siRNA oligos in presence/absence of Bafilomycin A1 (400 nM, 5 h). LC3-II/GAPDH ratio is displayed relative to control. (**h**) Cathepsin L activity in HeLa cells treated as in **e**. Bafilomycin A1 was positive control (400 nM, 3 h). Bars—means±s.d. (*n*=3; ***P<0.001, **P<0.01, NS, not significant; two-tailed *t*-test). (**i**) Number and size of GFP dots (autophagosomes) and mRFP-only dots (autolysosomes). HeLa cells stably expressing mRFP-GFP-LC3 were treated with two rounds of siRNA (smart-pool PFD6 and two individual oligos), then seeded in a 96-well plate and subjected to Cellomics visualization analysis. Graphs show the results for one representative experiment: 10 wells of 100–200 cells each were analysed per sample. Similar data was seen in other two independent experiments. Bars—means±s.e.m. (*n*=10; ***P<0.001, **P<0.01, *P<0.05; two-tailed *t*-test).

As these data show that actin depolymerization mirrors the lysosomal and autophagy defects seen in CCT-depleted cells, perturbations in the actin cytoskeleton dynamics may be mechanistically sufficient to explain the block in autophagosome degradation caused by CCT loss-of-function. Indeed, knockdown of individual CCT subunits in cells exposed to latrunculin A, further supported the importance of actin dysfunction as a mediator of the CCT-deficiency autophagy–lysosome phenotype, as latrunculin A failed to further increase basal LC3-II levels (Fig. 4a,b) or reduce cathepsin L activity (Fig. 4c,d) in CCT5-depleted cells. To reinforce the role of actin cytoskeleton in CCT-mediated autophagy, we next investigated the role of prefoldin (PFD) in the autophagy–lysosome pathway. Knockdown of prefoldin (depletion of any of the prefoldin subunits is enough to impair the PFD complex activity), the co-chaperone that delivers actin and tubulin to the TRiC complex for their final folding, recapitulated the major defects caused by CCT depletion or treatment with actin depolymerizing drugs (Fig. 4e–i). PFD6 knockdown reduced both autophagosome degradation and flux (quantified as LC3-II levels on western blot—Fig. 4e–g, or as number/size of yellow vesicles in HeLa cells stably expressing the mRFP-GFP-LC3 reporter—Fig. 4i) and cathepsin L activity (Fig. 4h). As in the case of CCT knockdown, latrunculin A failed to further reduce cathepsin L activity in PFD6 knockdown cells (Fig. 4h), reinforcing the role of actin in the autophagy–lysosome impairment found in both CCT- and prefoldin-deficient conditions. Thus, CCT knockdown appears to block the autophagy flux mainly through actin cytoskeleton-dependent mechanisms.

### CCT restricts mutant HTT accumulation via autophagy

**Figure 5:**
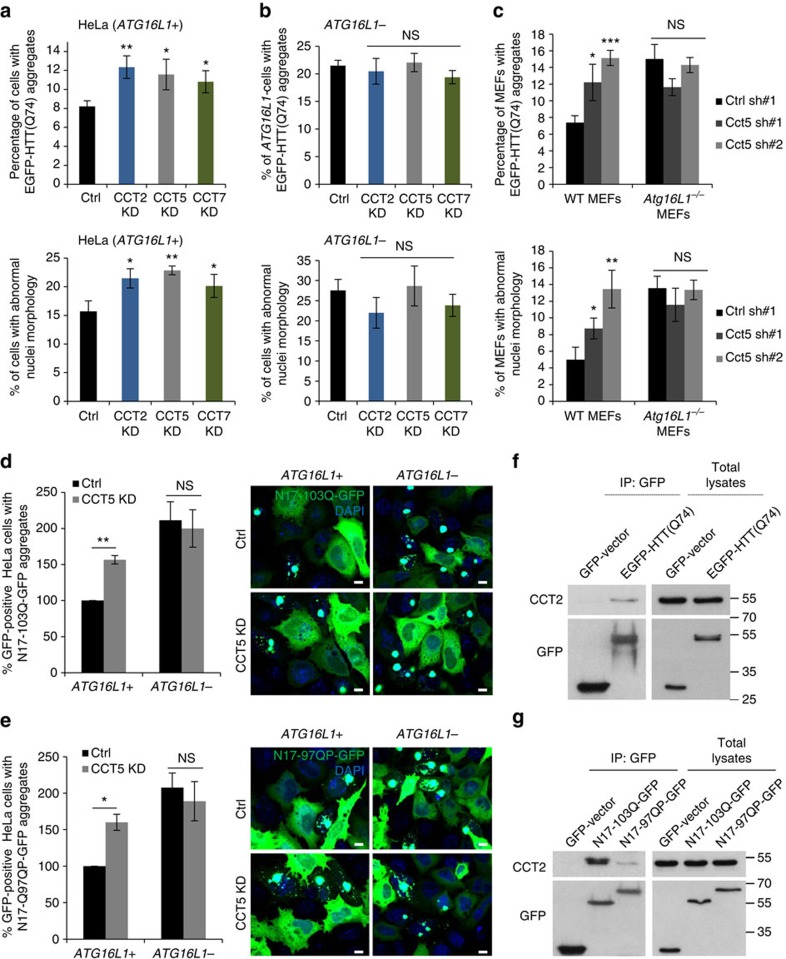
CCT depletion increases PolyQ aggregation consequently to reduced autophagic clearance. (**a**) EGFP-HTT(Q74) aggregation and cell toxicity in CCT-depleted HeLa cells. Upper: HeLa cells (*ATG16L1+*) were seeded on coverslips in triplicates, transfected with siRNAs targeting control or individual CCT subunits, followed by 1 μg of EGFP-HTT(Q74) overexpression. Bottom: percentage of nuclei with morphological changes (fragmented or condensed nuclei). Bars—means of percentages of cells with aggregates±s.d. (*n*=3; **P<0.01; *P<0.05; two-tailed *t*-test). Similar results were observed in four independent experiments. See Supplementary Fig. 9b for representative images. (**b**) EGFP-HTT(Q74) aggregation and cell toxicity in *ATG16L1−* CRISPR/Cas9 HeLa cells. The experiment was performed in parallel with **a**. Bars—means of percentages of cells with aggregates±s.d. (*n*=3; NS, not significant; two-tailed *t*-test). Similar results were observed in four independent experiments. See Supplementary Fig. 9b for representative images. (**c**) EGFP-HTT(Q74) aggregation and cell toxicity in WT and *Atg16L1*^*−/−*^ MEFs. Cells were seeded on coverslips in triplicates, transduced with lentiviral particles targeting control or the mouse Cct5 subunit (two independent shRNAs) and transfected with EGFP-HTT(Q74). Bars—means of percentages of cells with aggregates±s.d. (*n*=3; ***P<0.001, **P<0.01, *P<0.05, NS, not significant; two-tailed *t*-test). Similar data was achieved in 3 independent experiments. See Supplementary Fig. 9a for representative images. (**d**) N17-103Q-GFP aggregation in CCT-depleted *ATG16L1+* and *ATG16L1−* HeLa cells. Representative images of N17-103Q-GFP aggregates are displayed on right. Bars—mean of the percentages of cells with aggregates±s.e.m. (*n*=4 independent experiments, each performed in triplicates; **P<0.01, NS, not significant; one sample *t*-test). (**e**) N17-97QP-GFP aggregation in CCT-depleted *ATG16L1+* and *ATG16L1−* HeLa cells. Representative images of N17-103Q-GFP aggregates are on right. Bars—mean of the percentages of cells with aggregates±s.e.m. (*n*=4 independent experiments, each performed in triplicates; **P<0.01, NS, not significant; one sample *t*-test). (**f**) CCT co-immunoprecipitation with EGFP-HTT(Q74). HeLa cells were transfected with either GFP-vector or EGFP-HTT(Q74) for 16 h and GFP-tagged proteins were pulled-down with GFP-Trap. (**g**) CCT co-immunoprecipitation with N17-103Q-GFP and N17-97QP-GFP. HeLa cells were transfected with GFP-vector, N17-103Q-GFP or N17-97QP-GFP for 16 h and GFP-tagged proteins were pulled-down with GFP-Trap. Note: N17-103Q-GFP does not have the proline-rich domain, while N17-97QP-GFP has both N terminus and proline-rich domains of htt exon 1.

CCT2/5/7 depletions increased the percentage of cells with mutant huntingtin exon 1 EGFP-HTT(Q74) (exon 1 of HTT with a 74-polyglutamine expansion fused at its N-terminal to EGFP) aggregates and cell death (scored by assessing proportion of cells with apoptotic nuclei, which correlates well with other assays and which we have used extensively^33^,^34^) in autophagy-competent HeLa (*ATG16L1+*) cells (Fig. 5a, both upper and bottom panels; Supplementary Fig. 8a). This phenomenon occurs when autophagy is compromised^1^,^35^, and presumably also if chaperone activity is impaired^10^,^11^.

Previous studies^10^,^11^,^36^ had assumed CCT regulates huntingtin aggregation by direct binding and modulation of its folding, suggesting that this chaperone directly regulates the aggregation of many intracellular disease-associated aggregate-prone proteins. However, our data suggested that autophagy insufficiency could also account for this phenomenon. Indeed, CCT depletion was unable to increase the percentage of cells with EGFP-HTT(Q74) aggregates or apoptotic nuclei compared with control in autophagy-deficient HeLa cells (*ATG16L1−*) where *ATG16L1* has been depleted using the CRISPR/Cas9 genome-editing technique to compromise autophagy (Fig. 5b, both upper and bottom panels; Supplementary Fig. 8b–d). The *ATG16L1−* HeLa cells depleted for various CCT subunits were exposed in parallel to 2 different concentrations of EGFP-HTT(Q74) plasmid (1 μg in Fig. 5b and 0.5 μg in Supplementary Fig. 8c). At both concentrations, CCT2/5/7 subunit depletions had a similar effect and caused no increase in the percentages of the cells with aggregates compared with control. Moreover, the transfections with different EGFP-HTT(Q74) plasmid concentrations gave a dose-dependent increase in the percentages of cells with aggregates in control and CCT subunit knockdown cells (∼20% compared with 10% when used 1 or 0.5 μg EGFP-HTT(Q74) concentration, respectively—see Fig. 5b; Supplementary Fig. 8c). This phenomenon is the same as we have observed previously^37^. Those findings were next confirmed in another cell line—mouse embryonic fibroblasts (MEFs), where lentiviral transduction of Cct5 shRNAs increased the number of cells with aggregates/apoptotic nuclei in wild-type cells (WT), but failed to do so in the autophagy incompetent *Atg16L1*^*−/−*^ MEFs (Fig. 5c; Supplementary Fig. 8e).

Our data exclude the interpretation that the system might be saturated in the autophagy null cells precluding a further increase in the aggregation/oligomerization levels. We previously reported that the proportion of autophagy null MEFs with polyglutamine aggregates can increase when protein clearance pathways other than autophagy are perturbed^38^. Our data above show that CCT inhibition has no effect in *ATG16L1−* cells transfected with 0.5 μg EGFP-HTT(Q74), yet obtained twice as many *ATG16L1−* cells with aggregates when they were transfected with 1 μg EGFP-HTT(Q74)—thus this system was not saturated (Fig. 5b; Supplementary Fig. 8c). We additionally confirmed that MG132 (a proteasome inhibitor) increases the aggregation in both *ATG16L1+* and *ATG16L1−* HeLa cells (Supplementary Fig. 8f,g). The experiment was performed following the same protocol as when we depleted individual CCT subunits. We further confirmed that MG132 also increased the percentage of cells with aggregates in the *Atg16L1*^*−/−*^ MEFs at two different concentrations to a similar extent (Supplementary Fig. 8h).

As these EGFP-HTT(Q74) constructs lack the first eight amino terminal residues of the protein, we also examined the effect of CCT depletion on the aggregation of two other well-characterized mutant huntingtin exon 1 fragments which have complete N termini and C-terminal GFP tags in both the autophagy-competent and -incompetent cells. This is important as the first 17 amino acids of HTT are exposed to post-translational modifications involved in modulating the HTT localization and its clearance by autophagy under ER stress conditions^39^,^40^,^41^. The percentages of cells with N17-97QP-GFP and N17-103Q-GFP aggregates followed the same pattern as previously described for EGFP-HTT(Q74) aggregates: increased percentage of cells with visible aggregates upon CCT5 depletion in autophagy-competent HeLa cells, but no additional effect in autophagy-incompetent cells (Fig. 5d,e; Supplementary Fig. 8i). Moreover, CCT2 co-immunoprecipitated with all these huntingtin-exon1 fragments: EGFP-HTT(Q74) (Fig. 5f), N17-97QP-GFP and N17-103Q-GFP (Fig. 5g).

Thus, these data suggest that autophagy is the main contributor to the excess EGFP-HTT(Q74) aggregation in CCT knockdown cells (proteasome activity was not affected by the CCT knockdown (Supplementary Fig. 8j)). As previously reported^35^,^36^, the morphological characteristics (as size and cellular localization) of EGFP-HTT(Q74), N17-97QP-GFP or N17-103Q-GFP aggregates in cells exposed to CCT2/5/7 siRNAs were indistinguishable from those in control cells, and also between autophagy -competent and -incompetent conditions (Supplementary Fig. 9a,b, related to Fig. 5a–e).

**Figure 6:**
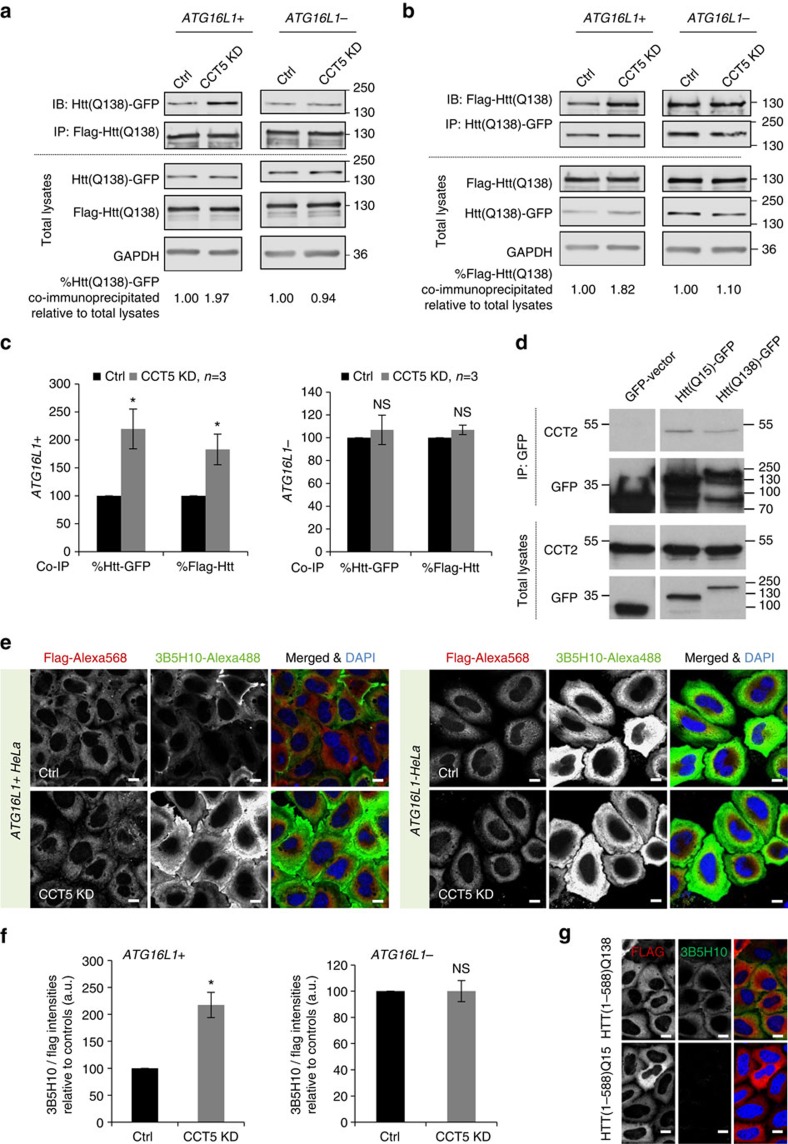
CCT depletion increases PolyQ oligomerization consequently to reduced autophagic clearance. (**a**,**b**) Oligomerization of mutant htt in CCT5-depleted Hela cells, both *ATG16L1+* and *ATG16L1−.* CCT5 depletion enhanced the amount of coimunoprecipitation of GFP-tagged mutant HTT(1–548) with Flag-tagged mutant HTT(1–588) —see left **a** and *vice-versa*—see right **b**, in autophagy competent HeLa cells, but failed to do so in the autophagy incompetent cells: *ATG16L1−* HeLa. (**c**) Quantification of mutant htt co-immunoprecipitation relative to total lysates/input from three independent experiments. The control condition was set to 100 and the bars represent the mean±s.d. (*n*=3; *P<0.05, NS, not significant; two-tailed sample *t*-test). See Methods. (**d**) CCT co-immunoprecipitation with the Htt(1–548)Q15 and Htt(1–548)Q138 constructs tagged with GFP at the C terminus. HeLa cells were transfected with GFP-vector, Htt(1–548)Q15 –GFP or Htt(1–548)Q138-GFP for 48 h and the GFP-tagged proteins were pulled-down using the GFP-Trap technology. (**e**) 3B5H10 staining in CCT5-depleted HeLa cells, both *ATG16L1+* and *ATG16L1−.* Scale bar throughout the panel is 10 μm. See Methods. (**f**) Quantification of 3B5H10 to Flag ratio signal intensities from three independent experiments. The bars represent the mean±s.d. (*n*=3; *P<0.05, NS, not significant; two-tailed sample *t*-test). (**g**) 3B5H10 antibody preferentially label cells overexpressing the pathogenic polyQ expansions in Htt(1–588). HeLa cells were transiently transfected with either Flag-tagged HTT(1–588)Q138 or Flag-tagged HTT(1–588)Q15 and immunostained with 3B5H10 antibody. Scale bar throughout the panel is 10 μm.

We next examined if CCT loss-of-function impacted the oligomerization of htt upstream of aggregate formation through autophagy and to which extent this would influence the formation of toxic intermediate species. As expected, knockdown of CCT5 subunit significantly increased the amount of co-immunoprecipitation of monomeric GFP-C-terminus-tagged mutant HTT(1–548) with Flag-N-terminus-tagged mutant HTT(1–588) and vice versa in HeLa cells, compared with control, as a measure of dimerization/oligomerization (Fig. 6a,b). Consistent with our aggregate counting data in Fig. 5a–e, knockdown of CCT5 had no effect on the mutant htt dimerization compared with control in autophagy-incompetent cells (Fig. 6a–c; Supplementary Fig. 9c). Additionally, treatment with MG132 was able to further increase the htt dimerization in *ATG16L1−* HeLa cells (Supplementary Fig. 9d), suggesting that the htt oligomerization is not saturated in the autophagy-incompetent cells. As expected, CCT2 co-immunoprecipitated with both the WT or mutant HTT(1–548) tagged to GFP at its C terminus (Fig. 6d).

To strengthen this observation, we next measured the abundance of pathogenic species with toxic conformations formed in the diffuse htt fraction of HeLa cells overexpressing Flag-tagged HTT(1–588) using the antibody 3B5H10, which recognizes a compact, two stranded conformation of polyQ in monomeric soluble mutant htt^42^,^43^. This monoclonal conformation-specific antibody recognizes soluble mutant htt species that predict neurodegeneration^42^,^43^. The cells exposed to CCT5 siRNA showed increased 3B5H10 intensity compared with control in *ATG16L1+* cells, but not in *ATG16L1−* HeLa cells (Fig. 6e,f), suggesting that also the pool of soluble toxic species follow the same pattern as the aggregation and oligomerization data. Fig. 6g validates that this antibody preferentially detects mutant htt species and not wild-type htt, as described previously^42^,^43^. Therefore, at this point, we concluded that the autophagy defect is the primary mechanism underlying the excess mutant htt oligomerization and aggregation when CCT2/5/7 functions are grossly impaired.

### CCT controls ATXN3 and p62 accumulation via autophagy

**Figure 7:**
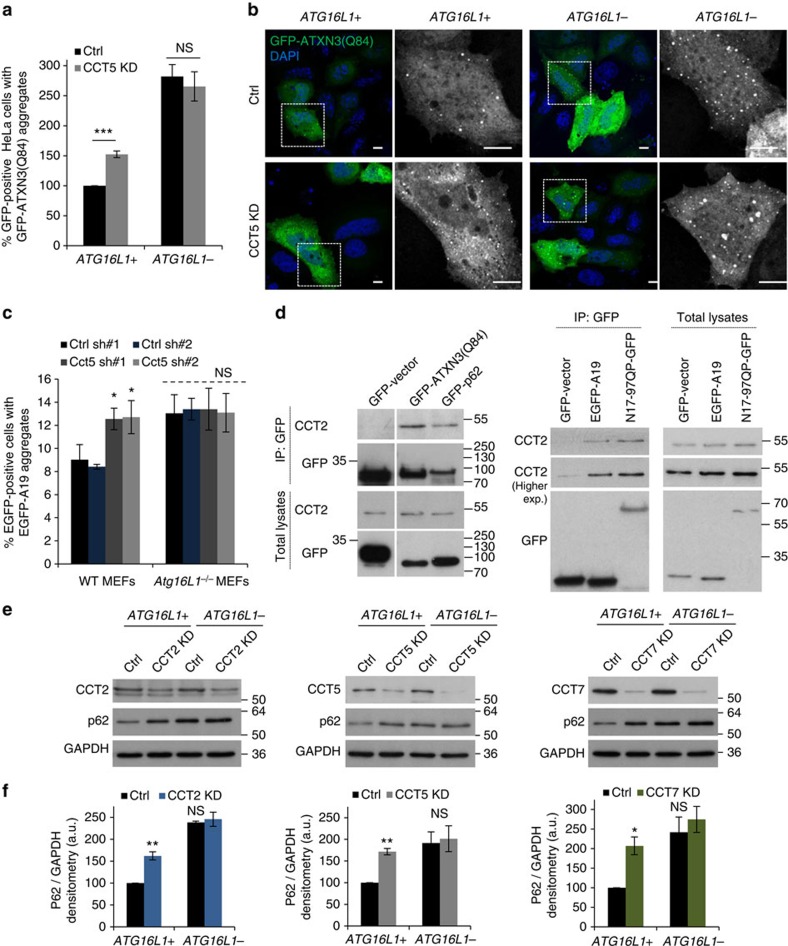
CCT depletion increases the accumulation of diverse autophagy substrates. (**a**) GFP-ATXN3(Q84) aggregation in CCT-depleted *ATG16L1+* and *ATG16L1-* HeLa cells. The bars represent the mean of the percentages of cells with aggregates±s.e.m. (*n*=4 independent experiments, each performed in triplicates; ***P<0.001, NS, not significant; one sample *t*-test). (**b**) Representative images of GFP-ATXN3(Q84) aggregates for the experiments performed in **a**. Scale bar throughout the panel is 10 μm. (**c**) EGFP-A19 aggregation in WT and *Atg16L1*^*−/−*^ MEFs. Cells seeded on coverslips in triplicates, were transduced with two different shRNA lentiviral particles targeting either control or the mouse Cct5 subunit and transfected with EGFP-A19. The bars represent the mean of percentages of cells with aggregates±s.d. (*n*=3; *P<0.05, NS, not significant; two-tailed *t*-test). Similar data was achieved in other two independent experiments. (**d**) CCT co-immunoprecipitation with the GFP-ATXN3(Q84), GFP-p62 (on the left ) and EGFP-A19, N17-97QP-GFP (on the right). HeLa cells were transfected with GFP-vector or the above mentioned proteins tagged to GFP for 16 h; the GFP-tagged proteins were pulled-down using the GFP-Trap technology. (**e**) Western blotting for p62. *ATG16L1+* and *ATG16L1−* CRISPR/Cas9 HeLa cells were transfected with siRNA targeting individual CCT subunits. GAPDH was used as loading control. (**f**) Quantification of p62 accumulation. *ATG16L1+* and *ATG16L1−* CRISPR/Cas9 HeLa cells were transfected with siRNA targeting individual CCT subunits as in (**e**). GAPDH was used as loading control. The bars represent the mean±s.d. (*n*=3; **P<0.01, *P<0.05, NS, not significant; two-tailed one sample *t*-test). Similar results were observed in three independent experiments.

We next questioned whether this observation could be extended to other autophagy substrates^44^,^45^ in multiple cell lines: HeLa, MEFs and primary cortical neurons. In autophagy-competent cells, CCT5 silencing enhanced the aggregation of transiently expressed GFP-tagged ATXN3(Q84) in HeLa cells (ataxin 3 protein with an expanded polyglutamine tract which causes spinocerebellar ataxia type 3 (ref. 46)) (Fig. 7a,b; Supplementary Fig. 10a), or long polyalanine tract EGFP-A19 (Fig. 7c) in MEFs and increased the levels of the endogenous autophagy substrate p62 in HeLa cells, primary cortical neurons and MEFs (Supplementary Fig. 10b–d). CCT2 or CCT7 depletion also increased the p62 levels in HeLa cells (Supplementary Fig. 10b). Similar to the mutant huntingtin-exon1 aggregation data, CCT2/5/7 knockdowns had no additional effect on the accumulation of these autophagy substrates in autophagy-deficient cells compared with controls (Fig. 7; Supplementary Fig. 10a,d). However, as p62 is also a proteasome substrate, we checked the p62 levels in *Atg16L1*^*−/−*^ MEFs when treated with MG132 and showed the autophagy-independent increase of p62 in this cell line (used as a positive control for increased p62 levels in autophagy incompetent cells—see Supplementary Fig. 10e). Thus, the p62 readout is not saturated. As in the mutant huntingtin-exon1 experiments, GFP-ATXN3(Q84)- or EGFP-A19-transfected cells did not show any differences in morphologies of the aggregates between the control or CCT5-depleted cells (Fig. 7b; Supplementary Fig. 10f). Interestingly, CCT2 strongly co-immunoprecipitated with all these additional autophagy substrates used: polyA, GFP-ATXN3(Q84) and GFP-p62 (Fig. 7d).

The autophagy inducers carbamazepine^47^,^48^ or Tat-Beclin1 peptide^49^, which enhance autophagosome biogenesis, significantly reduced the percentage of control cells with mutant htt aggregates. These inducers had no effects on the percentage of *ATG16L1*-null cells with mutant htt aggregates, thus these phenomena are autophagy-dependent. These inducers also had no effect on the percentage of CCT5-knockdown cells with mutant htt aggregates (Supplementary Fig. 10g). This is entirely consistent with our model suggesting that the predominant effects of CCT depletion on autophagy is at the level of the lysosome. In this scenario, induction of autophagosome biogenesis will be unable to significantly modulate degradation of substrate, as the block in the pathway is downstream of autophagosome formation. Furthermore, these data underscore the impact of the autophagy–lysosome pathway defect in CCT depletion, since if the autophagy–lysosome defect were mild or if the predominant effect of the individual CCT subunits depletions on the aggregation were via an autophagy–lysosome-independent mechanism, then autophagy induction would be able to reduce the levels of mutant huntingtin aggregation.

As lysosomal function is important for degradation of both autophagic and endocytic cargos and as depletion of individual CCT subunits reduces lysosomal activity (by increasing the lysosomal pH and impairing the trafficking of lysosomal enzymes as Cathepsins D and L), we next examined whether CCT5 depletion caused the accumulation specific endocytic cargos. Indeed, the levels of IGF1R, EGFR and β1 integrin increased in CCT5 depleted HeLa cells (Supplementary Fig. 11a). We confirmed that EGFR degradation kinetics (see Methods) was significantly retarded down in CCT5 knockdown cells, suggesting that the endocytic-lysosomal degradation route is perturbed (Supplementary Fig. 11b). However, we cannot exclude the possibility that these proteins could be clients of CCT/TRiC, thereby preventing proper refolding and reducing clearance.

### CCT depletion blocks autophagosome degradation in *Drosophila*

**Figure 8:**
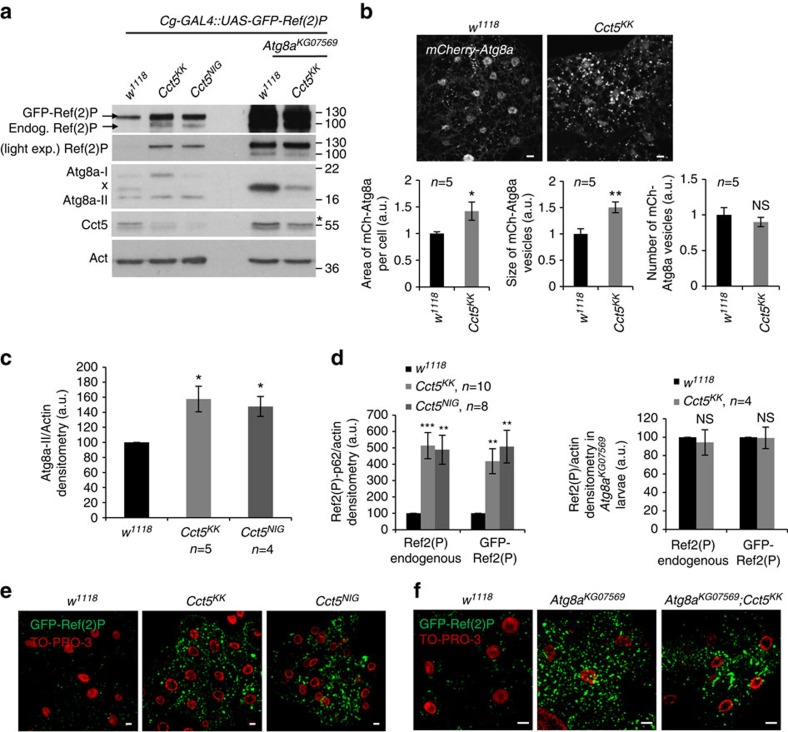
The increase of p62 levels and aggregation after *Cct5* depletion in *Drosophila* is autophagy dependent. (**a**) Biochemical assessment of autophagy upon *Cct5* knockdown in fat body of third instar larvae expressing GFP-Ref(2)P (*Cg-GAL4 UAS-GFP-Ref(2)P*) and either the RNAi for *Cct5* alone (using either *Cct5*^*KK*^ or *Cct5*^*NIG*^) or *Cct5*^*KK*^ in *Atg8a*^*KG07569*^ mutant background. VDRC *w*^*1118*^ (stock number 60100) was used as background control. Three larvae were lysed, and equal amounts of proteins for each control and test genotype were used for western blot analysis. Both Atg8-II and Ref(2)P (both GFP-tagged and endogenous) levels increased upon *Cct5* knockdown, while *Cct5* knockdown did not obviously influence Ref(2)P levels in *Atg8a* mutant fat bodies. x—non-specific band. Actin—loading control. See also Supplementary Fig. 12b. (**b**) Representative confocal images of fat body cells from third instar larval progeny of *Cg-GAL4 UAS-mCherry-Atg8a* crossed to either VDRC *w*^*1118*^ (stock number 60100, background control) or *Cct5*^*KK*^. Bars—mean±s.e.m., five larvae per each condition, >25 cells per larva. Size of mCherry-Atg8a-positive vesicles increased in *Cct5-*depleted cells, suggesting defective autophagosome degradation. (*n*=5; NS, not significant, *P<0.05; **P<0.01; two-tailed *t*-test). Scale bar, 10 μm. (**c**) Atg8a-II levels in third instar larval fat bodies upon knockdown of *Cct5* using *Cg-GAL4*, as in **c**. Bars—means±s.e.m., control ratio set to 100 (*P<0.05; two-tailed one sample *t*-test). (**d**) Quantification of GFP-tagged and endogenous Ref(2)P levels in third instar larval fat bodies upon knockdown of *Cct5* using *Cg-GAL4* as in **c**, either alone (top graphs), or in an *Atg8a* mutant background (*Atg8a*^*KG07569*^*;Cct5*^*KK*^, bottom graphs ). Bars—means±s.e.m., control ratio set to 100 (NS, not significant, **P<0.01, ***P<0.001; two-tailed one sample *t*-test). (**e**) Representative confocal images showing GFP-Ref(2)P in fat body of *Cct5* knockdown (using *Cg-GAL4*) third instar larvae. GFP-Ref2(P)-positive aggregates accumulate in *Cct5-*depleted cells. TO-PRO-3 stains the nuclei. Experiment was repeated with similar results (*n*>3). Scale bar, 10 μm. (**f**) Representative confocal images showing GFP-Ref(2)P in larval fat body cells co-depleted for both *Atg8a* and *Cct5* (*Atg8a*^*KG07569*^*;Cct5*^*KK109505*^). *Cct5* knockdown did not obviously enhance the GFP-Ref(2)P phenotype of *Atg8a* mutants. Experiment was repeated with similar results. TO-PRO-3 stains the nuclei. Scale bar, 10 μm.

We further explored these phenomena *in vivo* using *Drosophila*, where we downregulated the CCT5 homologue, *Cct5*, using two independent RNA interference (RNAi) lines (*Cct5*^*KK*^ or *Cct5*^*NIG*^, whose sequences do not overlap) or the CCT7 homologue, *Cct7*, using the *Cct7*^*KK*^ RNAi line (full genotypes of RNAi lines are in Methods). Fat body cells of male third instar larvae (L3) depleted of *Cct5* or *Cct7* RNAi showed consistent increases in the endogenous Atg8a-II (orthologue to the human LC3-II) levels and in size of mCherry-Atg8a vesicles compared with controls (Fig. 8a–c; Supplementary Fig. 12a). Additionally, levels of both endogenous and GFP-tagged Ref(2)P, the *Drosophila* p62 orthologue, were increased 5-fold in *Cct5-* or *Cct7*-knockdown flies, consistent with defective autophagic clearance (Fig. 8a,d,e; Supplementary Fig. 12a–c). However, in autophagy-defective *Atg8a*^*KG07569*^ larval fat bodies, RNAi depletion of either *Cct5* or *Cct7* did not increase the levels of both GFP-tagged and endogenous p62 levels compared with control (Fig. 8a,d,f; Supplementary Fig. 12a). At a macroscopic scale, both *Cct*-depleted and *Atg8a*^*KG07569*^ larvae showed a necklace-like phenotype in the fat body, where regions with bigger Ref(2)P aggregates alternated with less bright ones (Supplementary Fig. 12d). The autophagy phenotype seen in *Cct5-* and *Cct7*-depleted larval fat body cells is similar to the one described in literature for V-ATP-ase depletion^27^, consistent with the mechanism described in mammalian cells. These data suggest that the Ref(2)P/p62 aggregation seen *in vivo* systems depleted for *Cct5* or *Cct7* is autophagy dependent (Fig. 8; Supplementary Fig. 12a).

## Discussion

The activity of the chaperonin CCT/TRiC is impaired in devastating neuropathies and in Alzheimer's disease and has been proposed to regulate the aggregation of proteins like mutant huntingtin. We therefore investigated and focused on loss-of-CCT-function experiments, as those probe for the physiological roles of the complex. Our findings identify important cross-talk between two key components of the proteostasis network, since CCT is essential for optimal lysosome activity, and if impaired, then the consequent reduction in autophagosome flux results in the accumulation, oligomerization and aggregation of various disease-relevant autophagy substrates.

Our results suggest that the autophagy–lysosome inhibition is largely mediated by defective folding of actin. Both actin dysregulation and CCT dysfunction cause impairment of lysosomal activity and reduce autophagosome formation. As the autophagosome-degradation impairment overwhelms any additional inhibition of autophagosome biogenesis in CCT-depleted conditions, we focused on studying and elucidating the mechanism behind LC3 accumulation and its consequences on protein degradation. Autophagy–lysosome compromise is sufficient to cause the accumulation and excess aggregation of proteins like mutant huntingtin, p62 and mutant ATXN3 (refs 35, 45, 50). Defects in the lysosomal compartment would be predicted to interfere with both autophagic and endocytic routes. Indeed, in addition to the accumulation of well-characterized autophagy substrates, we also noticed an increase in endocytic cargos like IGF1R, EGFR and B1 integrin and slowed kinetics of EGFR degradation. However, we cannot exclude any direct chaperoning effect on CCT on these proteins which may also influence their fates.

CCT1 was previously shown to interact and sequester the N17-huntingtin exon 1 fragments^10^,^11^,^12^ as well as polyQ stretches^51^. Here we confirmed that CCT2 interacted with huntingtin fragments, polyA stretches, mutant ATXN3 and p62. It is not clear if the htt-CCT2 interaction is dependent on the N17 amino acids of htt, like CCT1 (refs 10, 11, 12, 14). It is interesting that the co-immunoprecipitation of CCT2 with the HTT-exon1 fragment is reduced in the presence of the proline-rich region compared with the HTT-exon1 without prolines (Fig. 5g)—this observation could be relevant to both the interaction and the effects described. Exon 1 of HTT also appears to assist in targeting huntingtin to autophagosomes and regulating HTT clearance by the lysosomal and proteasome pathways^39^,^40^,^41^. In future work, it would be interesting to evaluate the impact of HTT N17 phosphorylation on the type of autophagy modulated by CCT (for example, selective versus bulk autophagy), as CCT and HTT may work together in autophagy and htt phosphorylation appears to modify its interactions^39^,^52^,^53^. This might be important as WT huntingtin also functions as scaffold for selective autophagy^44^,^54^.

CCT was previously shown to interact with various components of the autophagic machinery involved in phagophore elongation, such as ATG16L1, ATG5, ATG10, ATG3 and TECPR1 (ref. 55). These may be relevant to the decrease in autophagosome biogenesis seen in CCT knockdown cells.

**Figure 9:**
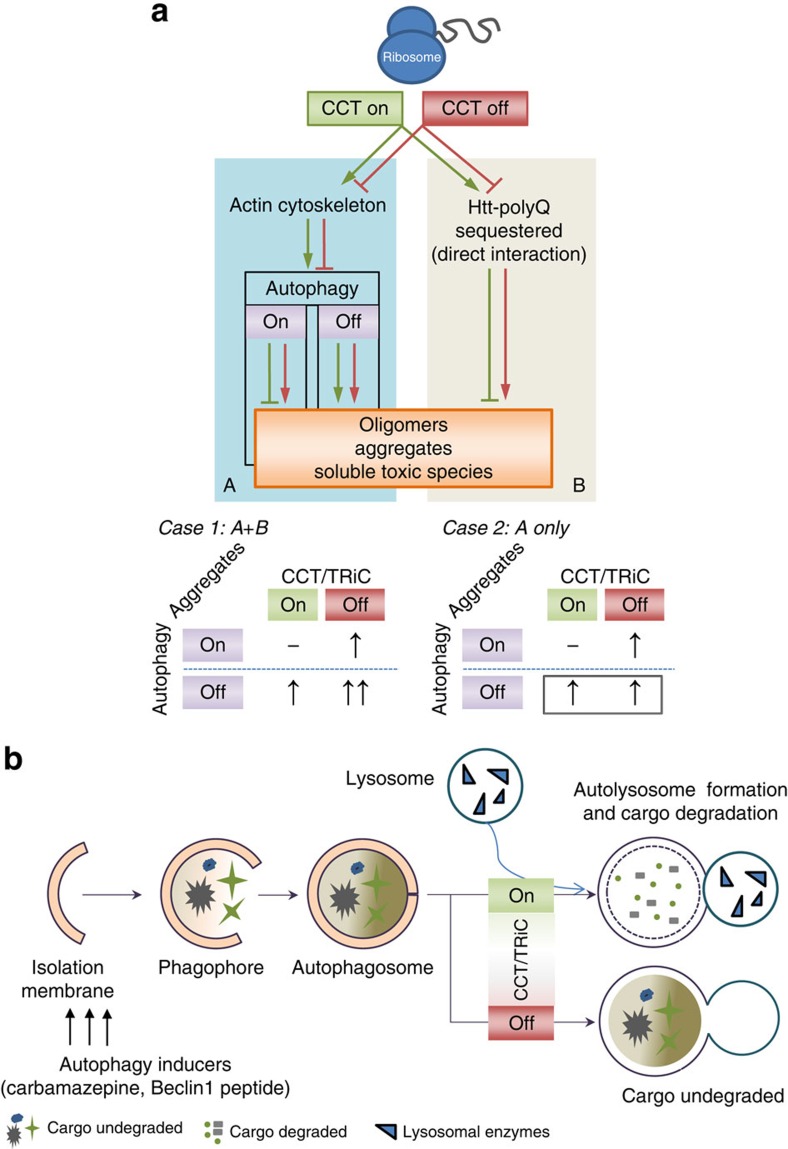
Model for the autophagy-dependent oligomerization/aggregation of mutant htt in CCT-depleted cells (**a**) Accumulation of oligomers, soluble toxic species and aggregates of mutant htt under CCT depletion may either be due to *case 1*: autophagy defects combined with reduced direct CCT interaction/sequestration of the mutant proteins, or *case 2*: autophagy inhibition only. In autophagy-competent cells, CCT depletion increases the mutant htt oligomerization and aggregation, compared with control. However in autophagy-incompetent cells, CCT depletion does not have additional effects on aggregate-prone protein accumulation, compared with control. This suggests that autophagy plays the major role in mutant htt aggregation in cell and *in vivo* models, and that any direct role of CCT in oligomerization of proteins like EGFP-HTT(Q74) is undetectable using the types of assays we have used. Since the readouts we have employed are not saturated, we conclude that proposed direct chaperoning effects of CCT on the oligomerization/aggregation of Q74 and related proteins is at best very small, compared with the autophagy effect of CCT compromise. (**b**) Schematic overview of autophagy-lysosome pathway defects under CCT depletion and subsequent impact on mutant proteins aggregation. Since the final step in autophagy–lysosome pathway is severely compromised, autophagy inducers acting at the level of autophagosome biogenesis (carbamazepine or Beclin1 peptide) are not able to impact on the aggregation phenotype.

Our data extend beyond the CCT–HTT interaction^10^,^11^,^12^,^51^, as we have seen similar interactions with other autophagy adaptors/cargos such as ATXN3, p62 or polyA tract that indeed accumulate in a similar autophagy-dependency pattern upon CCT depletion. The effects of the CCT depletion on the aggregation and oligomerization of mutant huntingtin fragment, polyalanine expanded proteins, mutant ATXN3 and p62 are primarily autophagy dependent, as opposed to the current assumption that these proteins are direct clients of physiological CCT activity and that this direct chaperone activity on mutant huntingtin is entirely responsible for its anti-aggregation properties (Figs 5 and 6). If the latter were critical, then one may expect that knockdown of CCT subunits would further enhance the aggregation/oligomerization/soluble toxic species accumulation of mutant huntingtin and related substrates in autophagy-deficient cells and *Drosophila* (see schema in Fig. 9a). Likewise, agents that induce autophagy at the level of autophagosome formation had no significant effects on the proportion of CCT-depleted cells with aggregates, which is what one would expect when there are major defects at the downstream lysosomal stages (Fig. 9b). Since autophagy activators would be expected to reduce the aggregation phenotype if CCT depletion acted only partly via the autophagy–lysosome pathway, or if the lysosomal defect still enabled some increased autophagy substrate flux upon induction of autophagosome biogenesis, these data reinforce the concept that the predominant consequence of CCT depletion on htt aggregation is by compromise of the autophagy–lysosome pathway. While we do not exclude that proteins like mutant huntingtin are direct CCT substrates or that overexpression of components of this complex may assist appropriate mutant huntingtin folding, our experiments suggest that loss of physiological CCT complex activity affects the mutant huntingtin, ataxin 3 and p62 accumulation, aggregation or toxicity mainly as a consequence of autophagy impairment, and that the contribution of any loss of direct endogenous chaperone complex activity on mutant huntingtin, p62 or ataxin 3 is minimum (undetectable) in our systems. We have not specifically tested CCT1 in our studies, and therefore cannot conclusively exclude that CCT1 acts in part through direct inhibition of htt aggregation. This is possible, since the ApiCCT1 domain alone is unlikely to be working through an alteration of CCT activity, as it lacks the domains responsible for the formation of the TRiC complex, having only the Apical client interaction domain^10^,^11^,^12^,^14^. However, our data are most relevant to the activity of the CCT complex as a whole.

As we used GFP-tagged constructs in most of our experiments, GFP tags may influence the degradation route options available to such proteins, for instance by increasing oligomerization and shifting flux from the proteasome to autophagy or by influencing phosphorylation events in htt that influence its clearance^52^,^56^,^57^. However, it is important to point out that the clearance of these GFP-fusions are autophagy-dependent as are the clearance of untagged mutant htt^58^, mutant ataxin 3 (ref. 59) and p62 (ref. 50).

Importantly, the accumulation and aggregation of proteins in autophagy-defective mouse brains is sufficient to cause neurodegeneration^60^,^61^, and decreased autophagy exacerbates the aggregation and toxicity of many intracytoplasmic aggregate-prone proteins, suggesting that the autophagy impairment resulting from CCT inhibition is sufficient to be a major driver of pathology. Therefore, pathological conditions with downregulated levels of CCT (for example Alzheimer's disease^6^) might manifest proteostasis disruption in both the chaperone and autophagy–lysosome systems. While this model is consistent with our toxicity data in cell culture models, it is likely that other direct clients of this chaperone will accumulate in misfolded states in such cells, which will also have likely deleterious consequences. Furthermore, the pathological consequences of autophagy impairment likely extend beyond the accumulation of specific aggregate-prone proteins to other phenotypes relevant to Huntington's disease and other neurodegenerative diseases, including reduced clearance of dysfunctional mitochondria leading to elevated levels of reactive oxygen species, and increased susceptibility to apoptotic insults^1^.

## Methods

### Antibodies

The antibodies used for western blot (WB) and immunofluorescence include: rabbit polyclonal anti-Calnexin (1:300 IF, Abcam), mouse monoclonal anti-Cathepsin D (1:200 IF, ab6313 Abcam), mouse monoclonal anti-Cathepsin L (1:500 WB, 611084 BD Bioscience), rat anti-Cathepsin L (1:1,000 WB, MAB9521 R&D Systems), goat anti-mouse Cathepsin L (1:1,000 WB, AF1515 R&D Systems), mouse monoclonal anti-GAPDH (1:5,000 WB, ab8245 Abcam), rabbit polyclonal anti-LC3 (1:3,000 WB, NB100-2220 Novus Biological), mouse monoclonal anti-LC3 (1:200 IF, clone 5F10 Nanotools), mouse monoclonal anti-LAMP1 (1:0 IF, clone H4A3 Developmental Studies Hybridoma Bank, University of Iowa), rabbit polyclonal anti-LAMP1 (1:200 IF, ab2417 Abcam), mouse monoclonal anti-tubulin (T9026 Sigma-Aldrich), 3B5H10 mouse monoclonal anti –polyglutamine (1:300 IF, P1874 Sigma-Aldrich), anti-Flag (1:1,000 WB, 1:300 IF, F1804 and F7425 Sigma-Aldrich), rabbit polyclonal anti-EGFR (1:250 WB, sc-03 Santa Cruz Biotechnology), rabbit polyclonal anti-IGF1R (1:250 WB, sc-713 Santa Cruz Biotechnology), mouse anti β1-integrin (1:1,000 WB, Clone 19/CD29 610467 BD Biosciences). The CCT antibodies were purchased from Sigma-Aldrich and used at 1:1,000 for WB:mouse monoclonal anti-CCT2 (WH001057M1), mouse monoclonal anti-CCT5 (WH0022948M1), rabbit polyclonal anti-CCT7 (HPA008425). Phalloidin-Alexa Fluor 546 from Invitrogen was used for F-actin staining.

Secondary antibodies for immunofluorescence were conjugates to Alexa Fluor 488, 568, 594 and 647 (Invitrogen). Anti-mouse and anti-rabbit HRP-conjugated secondary antibodies were from GE Healthcare; LICOR antibodies: anti-mouse 680 and anti-rabbit 800.

### Plasmids and siRNAs

Huntington's disease gene exon 1 fragment with 74 poly-Q repeats in pEGFP-C1 (Clontech; EGFP-HTT(Q74)) was previously characterized^37^. The poly(A) stretch of 19 repeats (A19) tagged with pEGFP (EGFP-A19) was previously described and characterized^35^. The N17-97QP-GFP and N17-103Q-GFP constructs^41^ were kindly provided by Prof. L.M. Thompson and Prof. J.S. Steffan (University of California, Irvine). The mutant HTT(1–588) tagged to Flag at its N terminus was generated by Dr S. Luo^62^, while the mutant HTT(1–548) tagged to GFP at its C terminus was a gift from Dr M.R. Hayden (University of British Columbia)^63^ and previously characterized by our laboratory^34^,^62^,^64^. GFP-p62 construct was a gift from Prof. Terje Johansen (University of Tromso, Norway)^50^. GFP-ATXN3(Q84) was kindly provided by Dr. Henry Paulson (Addgene #22123)^65^. On-Target plus SMART pool siRNA against human CCT2 (L-020107-00-0005), CCT5 (L-012797-00-0005), CCT7 (L-020115-00-0005), PFD6 (L-020630-02-0005 for smart-pool and LQ-020630-02-0002 for individual oligos) were purchased from Dharmacon (siRNA nucleotide sequences are provided in Supplementary Table 1).

### Reagents

Bafilomycin A1 (Millipore) was resuspended in dimethyl sulphoxide (DMSO from Sigma-Aldrich) and used at a saturating concentration for autophagosome degradation of either 400 nM for 4–6 h or 200 nM left overnight. As Bafilomycin A1 is dissolved in DMSO, all conditions without BafA1 received an equivalent volume of DMSO. Latrunculin A (L5163) and Cytochalasin D (C8273) were purchased from Sigma-Aldrich, resuspended in DMSO and used at a final concentration of 1 μM in the culture media. MG132 (M8699) and Carbamazepine (C4024) were purchased from Sigma-Aldrich. Tat-Scrambled and Tat-Beclin1 were purchased from Protein Chemistry Technology Core, UT Southwestern Medical Center.

### Cell culture

HeLa cells and MEFs were grown in DMEM (D6546 Sigma-Aldrich) supplemented with 10% fetal bovine serum, 100 U ml^−1^ penicillin–streptomycin, 2 mM L-glutamine (all from Sigma-Aldrich) at 37 °C and 5% CO_2_, humidified atmosphere. HeLa cells (source ATCC) were authenticated by STR profiling. HeLa cells stably expressing either mRFP-GFP-LC3 or GFP-LC3 were grown in the same media supplemented with 600 μg ml^−1^ of G418 (1181-031 Invitrogen). WT and *Atg16L1*^*−/−*^ MEFs^66^ were kindly provided by Prof. Tamotsu Yoshimori (Osaka University).

Mouse primary cortical neurons were cultured as described by Jimenez-Sanchez *et al*.^34^ All the cell lines were routinely tested for mycoplasma contamination.

### CRISPR/Cas9 HeLa generation line for *ATG16L1*

HeLa CRISPR/Cas9 *ATG16L1* knockout cell lines were generated by double nickase to avoid off target activity following the methods described in Ran *et al*.^67^ and Ran *et al*.^68^ Briefly, guide pairs nicking the first exon of *ATG16L1* (192–211 and 146–166) were designed using the Zhang lab CRISR design tool (http://www.genome-engineering.org/crispr/) and cloned into the pSpCas9n(BB)-2 A-GFP (PX461: Addgene 48410). gRNAs with overhangs were annealed as described in Ran *et al*.^67^ without phosphorylation and PX461 was digested with BbsI for 1 h followed by ligation in the same step for another hour (based on ‘ELAN' method described by Cost and Cozzarelli^69^). HeLa cells were transfected with both gRNAs simultaneously or with the equivalent amount of empty PX461. Two days after transfection, GFP-positive cells were single-cell sorted into 96-well plates using fluorescent-activated cell sorting (FACS). Clones were expanded and those where ATG16L1 was undetectable by western blotting were selected. Empty Cas9 plasmid transfected cells subjected to the same procedure were used as WT HeLa control cells.

### mRFP-GFP-LC3 neurons

All studies and procedures were performed under the jurisdiction of appropriate Home Office Project and Personal animal licenses and with local Ethics Committee approval. mRFP-GFP-LC3 was subcloned by PCR from pmRFP-EGFP-rLC3 (gift from T. Yoshimori) into pCAGGS (gift from J. de Belleroche) which drives ubiquitous expression of the transgene under the control of a chicken beta-actin promoter and cytomegalovirus enhancer. The vector was digested with HindIII and SpeI to remove the DNA backbone and the fragment purified by gel extraction and used for microinjection into hybrid B6CBA oocytes as described in Behringer (ref. 70). Founders were identified by PCR from ear biopsies and crossed to C57BL6 mice to obtain F1s. mRFP-GFP-LC3 protein expression level was assessed by western blotting in brain, muscle and tissue and by cryosectioning of fresh frozen tissue and direct observations of fluorescence levels. Line 1 was selected for further study due to its good levels of protein expression and clear fluorescence in brain. This line was maintained by further backcrossing to C57BL6 mice.

For primary mRFP-GFP-LC3 cortical neurons, transgenic mice were crossed with C57BL6 mice, at E16.5 gestation females were sacrificed and embryos were harvested. Cortices from all embryos (regardless of genetic status) and combined to create mixed cultures. Neurons were cultured as described by Jimenez-Sanchez *et al*.^34^ At DIV9-10 mRFP-GFP-LC3 neurons are imaged using confocal microscopy (63 × NA 1.4 Plan Apochromat oil immersion lens; Carl Zeiss LSM710 and LSM780).

### siRNA transfection

In all the siRNA transfection experiments, HeLa cells were seeded in six-well plates one day before and transfected with 100 nM of the indicated siRNA using Lipofectamine 2000 (Invitrogen). On the second day the cells were transfected again with 50 nM of siRNA. At 48 h post transfection the cells were split and reseeded in 6-well plates according to the experiment's requirement. Cells were further cultured in full medium for 48 h. HeLa cells stably expressing the mRFP-GFP-LC3 reporter were transfected following the protocol above, except that at 72 h post transfection the cells were reseeded in 96-well plates.

### Lentiviral transduction

The following individual shRNAs containing pLKO.1 vectors targeting the mouse Cct5 were purchased from The RNAi Consortium (TRC): Cct5 sh#1 (TRCN0000120433) and Cct5 sh#2 (TRCN0000120436). Ctrl sh#1 (lentiviral pLKO.1 Empty Vector Control) was obtained from TRC (RHS4080) and Ctrl sh#2 (scramble shRNA vector) was previously described by Jimenez-Sanchez *et al*.^34^, available at Addgene, code 1864.

To generate the viruses, we cotransfected actively growing HEK293T with the target shRNA, the second generation packaging plasmid containing gag, pol and rev genes and the envelope plasmid (VSV-G expressing plasmid, pMD2.G) using the TransIT-LT1 transfection reagent (MIR 2300/5/6 Mirus Bio). We harvested the virus containing the supernatants at 48, 72 and 96 h post-transfection and concentrated them by centrifugation in 15 ml Beckman tubes at 30,000 r.p.m. for 90 min at 4 °C. The MEFs were plates in 6-well plates and transduced with 40 μl of the 100 × viruses stock on the following day. Forty-eight hours post-transduction the cells were split and reseeded on coverslips for EGFP-HTT(Q74) overexpression. Primary neurons were seeded in 12-well plates and transduced with 20 μl of the 100 × viruses stock on day 4 of culture. The media was replaced on the following day.

### Immunofluorescence and microscopy

For double immunostaining studies of LC3/LAMP1, cytoskeleton or Cathespin D/LAMP1 the cells were fixed and permeabilized in −20 °C cold methanol for 4 min. The cells were rinsed several times in phosphate-buffered saline (PBS) and blocked for 1 h in 0.3% BSA (BP1605-100 Fischer Scientific). For immunostaining of LAMP1 (mouse antibody) and Cathepsin D/Calnexin, the cells were fixed in PFA 4% for 5 min, permeabilized with Triton 0.1% for 10 min and then blocked in 3% BSA for 1 h. For Cathepsin D staining, the cells were incubated in primary antibody buffer for 2 h at 25 °C. For all other immunostaining, the coverslips were left in primary antibody at 4 °C overnight. After a secondary conjugation with Alexa Fluor antibodies for 90 min, the coverslips were mounted in Prolong Gold Antifade reagent (with DAPI; P-36931 Invitrogen). Coverslips were examined with a confocal microscope (63 × NA 1.4 Plan Apochromat oil immersion lens; Carl Zeiss LSM710, LSM780 and LSM880). When necessary, the images were exported in Photoshop (Adobe) and equal adjustments were made for all the images from all treatment groups.

### 3B5H10 antibody staining

The 3B5H10 antibody was previously characterized as detecting a toxic conformation of htt^42^,^43^. *ATG16L1+* and *ATG16L1−* HeLa cells were exposed to two rounds of control or CCT5 siRNAs and seeded in 12 well plates. The following day, the cells were transfected with 0.4 μg of Flag-tagged-HTT(1–588) and 5 μl of TransIT-2020 (Mirus) for 18 h, then fixed in PFA 4% for 5 min, blocked for 1 h in 5% goat serum and incubated with primary antibodies at 4 °C overnight: 3B5H10 (mouse monoclonal, Sigma-Aldrich) and rabbit anti-Flag (Sigma-Aldrich). After a secondary conjugation with Alexa Fluor antibodies for 90 min (anti-mouse Alexa 488 and anti-rabbit Alexa 568), the coverslips were mounted in Prolong Gold Antifade reagent (with DAPI; P-36931 Invitrogen). Coverslips were examined with a confocal microscope (63 × NA 1.4 Plan Apochromat oil immersion lens; LSM780). Eight to ten random fields of 6–10 cells each were imaged per condition. The signal intensities were quantified using ImageJ. The acquisition settings were identical among all the conditions examined.

### Quantification of colocalization

For quantifying the colocalization of different proteins, at least 10 fields of 3–5 cells per field were taken for each sample per experiment. Each experiment was performed at least twice while the exposure settings were unchanged during acquisition of various samples. Images were examined with Volocity software and Pearson's coefficients were used for quantifying the colocalization. Statistical analysis was performed using Student's *t*-test.

### Quantification of cell toxicity and polyQ aggregation

EGFP-HTT(Q74), N17-103Q-GFP, N17-97QP-GFP and GFP-ATXN3(Q84) aggregation were monitored with a fluorescence microscope. The counting method has been previously described^35^. When using EGFP-HTT(Q74), at least 250 cells were counted per coverslip and the proportion of cells with at least one aggregate was scored as a percentage of the total number of transfected cells. The apoptotic cell death was assessed by measuring the percentage of nuclei with morphological changes (fragmented or condensed nuclei). The experiments were performed without knowing the identity of the slides at least three times in triplicates.

For analysing the N17-103Q-GFP, N17-97QP-GFP aggregation, at least 300 cells were counted per coverslip and the proportion of cells with at least one aggregate was scored as a percentage of the total number of transfected cells. For analysing the GFP-ATXN3(Q84) aggregation at least 400 cells were counted per coverslip.

### Cell lysis and western blot analysis

Cells were washed twice in cold PBS and lysed in RIPA buffer (150 nM NaCl, 1% NP40, 0.5% NaDoc, 0.1% SDS, 50 mM Tris, from Sigma-Aldrich) and a protease inhibitors mix from Roche Diagnostics). The samples were kept 30 min at 4 °C to ensure complete lysis and then centrifuged at 16,200 *g* for 15 min to pellet the insoluble membranes. The protein levels from supernatant were quantified using an assay based on the Bradford method, the BioRad Protein Assay Kit II (BioRad) and normalized for loading. Cell lysates were further diluted in 2 × Laemmli Sample Buffer (161-0737 BioRad), boiled for 7–10 min at 100 °C and subjected to an SDS–PAGE separation, followed by transfer on PDVF membranes using the BioRad mini gel system. The membranes were blocked in 6% milk in 0.1% Tween in PBS for 1 h and incubated with primary antibody at 4 °C overnight. Incubation with secondary antibody for 90 min was followed by protein visualization either with the ECL detection kit (GE Healthcare) or directly on a LICOR machine. Proteins levels were quantified using ImageJ software.

### Co-immunoprecipitation (dimerization) assay

This assay was previously described^34^,^62^. *ATG16L1+* and *ATG16L1−* HeLa cells were exposed to two rounds of control or CCT5 siRNAs and seeded in 6-well plates. For Flag immunoprecipitation, cells were transfected with 1.5 μg of GFP-tagged-HTT(1–548) and 1 μg of Flag-tagged-HTT(1–588) combined with 5 μl of TransIT-2020 (Mirus) for 24 h, and lysed in Buffer A, containing 20 mM Tris pH 7.4, 150 mM NaCl, 2 mM MgCl_2_, 0.5% NP40, protease inhibitors mix and phosphatase inhibitors from Roche Diagnostics. For GFP immunoprecipitation, cells were transfected with 1 μg of GFP-tagged-HTT(1–548) and 1.5 μg of Flag-tagged-HTT(1–588) combined with 5 μl of TransIT-2020 (Mirus) for 24 h, and lysed in Buffer B, containing 10 mM Tris pH 7.4, 150 mM NaCl, 1 mM EDTA pH8, 1% Triton, protease inhibitors mix and phosphatase inhibitors from Roche Diagnostics. Six hundred micrograms total protein per condition were incubated with primary either anti-Flag M2 (Sigma-Aldrich) or anti-GFP (rabbit polyclonal Clonotech) at 4 °C overnight. The immune-complexes were cleared with magnetic Protein G Beads (Invitrogen). Input lysates were run simultaneously with the IP samples on 10% polyacrylamide gels and visualized with LICOR.

### GFP-Trap method

GFP-Trap immunoprecipitations were performed using the GFP-Trap_MA beads (Chromotek gtma-20) following the manufacturer's instructions.

### Lysosomal pH and Cathepsin L activity

One day before live cell imaging, HeLa cells were seeded in 35 mm petri dishes with 14 mm microwells (P35G-1.0-14-C MatTek). LysoSensor Yellow/Blue DND-160 (L-7545 Molecular Probes) probe was added at 1.5 μM in full medium for 20 min, removed, then the cells washed twice and visualized at a confocal microscope. Primary cortical neurons were seeded in the MatTek live-cell dishes, treated with shRNA lentiviral particles (standard protocol: 20 μl of the 100 × viruses stock on day 4, media changed in the following day and cultured for another 5–6 days) and probed for LysoSensor Yellow/Blue at 1.5 μM in full medium for 5–10 min. The fluorescence intensities in the blue and yellow channels were quantified in ImageJ.

The lysosomal protease activity was measured using the Cathepsin L Activity Kit (65306 Abcam) following the manufacturer's instructions.

### Proteasome activity

HeLa cells were seeded in 6-well plates and the assay was performed in triplicates. First, the cells were washed twice with PBS, then collected in 1.5 ml Eppendorf tubes and centrifuged at 2,000 r.p.m. for 5 min at 4 °C. The cellular pellet was resuspended in 100 μl 50 mM Tris, pH 7.4, I mM DTT and sonicated twice for 10 s. The cellular debris was removed by centrifugation (13,000 r.p.m., 10 min, 4 °C), then the supernatant was collected in a separate tube. For measuring the chymotrypsin-like activity of the proteasome, 25 μg proteins were used per reaction together with 100 μM of Suc-LLVY-ACM (Enzo, BML-P802-0005). The activity was measured for 1 h at 37 °C at 5 min interval using a plate reader with Ex. 380 nm and Em. 460 nm.

### EGFR degradation assay

HeLa cells were serum starved (DMEM with 0.2% fetal bovine serum) for 4 h before loading with the receptor ligand EGF (100 ng ml^−1^) in the presence of cyclohexamide (CHX, 40 μg ml^−1^) for 30 min. After, the cells were washed twice with PBS, and further incubated in serum-free media in the presence of cyclohexamide for another 1 or 2 h. At ‘time 0' the cells were lysed immediately after the PBS wash. For each individual time point we normalized the data to ‘time 0' (EGF+; CHX+) and used two-tailed *t*-test to assess the protein level differences between Ctrl- and CCT5-depleted cells. Two ways ANOVA was performed to assess the overall significance of EGFR degradation between the control and CCT5 knockdown cells.

### *Drosophila* stocks and genetics

To check the effect of *Cct* subunits downregulation on the autophagy substrate Ref(2)P/p62, flies of the genotype *w; Cg-GAL4 UAS-GFP-Ref(2)P*^71^ were crossed to UAS-RNAi lines for *Cct5* (*Cct5*^*K108095(v109505)*^, labelled *Cct5*^*KK*^, or *Cct5*^*NIG.8439R*^, labelled *Cct5*^*NIG*^ - the sequences of these RNAi lines do not overlap) or *Cct7* (*Cct7*^*KK101540(v108585)*^, labelled *Cct7*^*KK*^ ) or to the appropriate background control (*w*^*1118*^, VDRC stock 60100). For assessing whether the phenotype due to *Cct5* and *Cct7* downregulation was autophagy dependent, virgins of genotypes *Atg8a*^*KG07569*^ (ref. 72)*, Atg8a*^*KG07569*^*; Cct5*^*KK*^or *Atg8a*^*KG07569*^*; Cct7*^*KK*^were crossed with males of genotype *w;Cg-GAL4 UAS-GFP-Ref(2)P,* and *Atg8a*^*KG07569*^*/Y* crawling third-instar larvae were selected for western blot or immunofluorescence. Otherwise virgins expressing *w;Cg-GAL4 UAS*-*mCherry-Atg8a* (ref. 73) were crossed with either *Cct5*^*KK*^ or *w*^*1118*^males. For western blots, the fat bodies of three larvae per genotype were dissected and lysed in Laemmli buffer. The following antibodies were used: rabbit polyclonal antibody anti-Ref(2)P (1:5,000 WB^74^), rabbit polyclonal antibody anti-Atg8a (1:5,000 WB^74^); mouse monoclonal anti-CCT5 (1:500 WB, WH0022948M1 Sigma-Aldrich), rabbit polyclonal anti-CCT7 (1:500 WB, HPA008425 Sigma-Aldrich) and rabbit polyclonal anti-actin (1:2,000 WB, A2066 Sigma-Aldrich). For immunofluorescence analysis, the fat bodies of 5 larvae per genotype were fixed in 4% formaldehyde for 30 min at room temperature (Sigma) and the DNA stained with TO-PRO-3 (1:1,500; Life Technologies). Pictures of the whole larvae were taken using a Leica MZ16F microscope connected to a Leica DFC340FX digital camera. The macroscopic images showing GFP-Ref2(P) aggregates in Extended Data Fig. 11e were taken with either a 2 × objective (whole larvae images) or an 8 × objective (enlarged images). Quantification of western blot bands or Atg8a vesicles (number, area and size per cell) was performed using ImageJ and the statistical analysis done as described above.

### Statistical analysis

Densitometry of western blot bands was performed using ImageJ. Each image was set to 8 bits format, inverted and the background was subtracted before measuring the band intensity in ImageJ. The graphs show the mean from either independent experiments or one representative experiment in triplicates. For each graph we specified whether the error bars are standard deviations (s.d.) or s.e. of the mean (s.e.m.). The *P* values for the densitometry were either determined with one sample *t*-test in Origin v8.1, where the control was initially set up to 100% (when the data from multiple experiments were combined) or two-tailed/one-tailed Student's *t*-test in Excel software, otherwise. For the polyQ aggregation representative experiments performed in triplicates, the *P* value was determined using the two-tailed *t*-test and the error bars represent the s.d. When multiple polyQ experiments were pulled together, the *P* value was determined using the one sample *t*-test and the error bars represent the s.e.m. For statistical comparisons we considered similar variance between the groups being compared. For colocalization experiments, we used Volocity software to determine the Pearson's coefficients and two-tailed *t*-test to compute the *P* value within one representative experiment. All experiments were performed at least twice, unless otherwise specified.

### Data availability

Authors can confirm that all relevant data are included in the paper and/or its supplementary Information files.

## Additional information

**How to cite this article:** Pavel, M. *et al*. CCT complex restricts neuropathogenic protein aggregation via autophagy. *Nat. Commun.* **7,** 13821 doi: 10.1038/ncomms13821 (2016).

**Publisher's note:** Springer Nature remains neutral with regard to jurisdictional claims in published maps and institutional affiliations.

## Supplementary Material

Supplementary Information Supplementary Figures.
