# Supplementary material for: CCT complex restricts neuropathogenic protein aggregation via autophagy
Source: Nat Commun. 2016 Dec 8;7:13821. doi: 10.1038/ncomms13821 (PMC5155164; doi:10.1038/ncomms13821)
Supplement: Supplementary Information — Supplementary Figures. [file ncomms13821-s1.pdf]

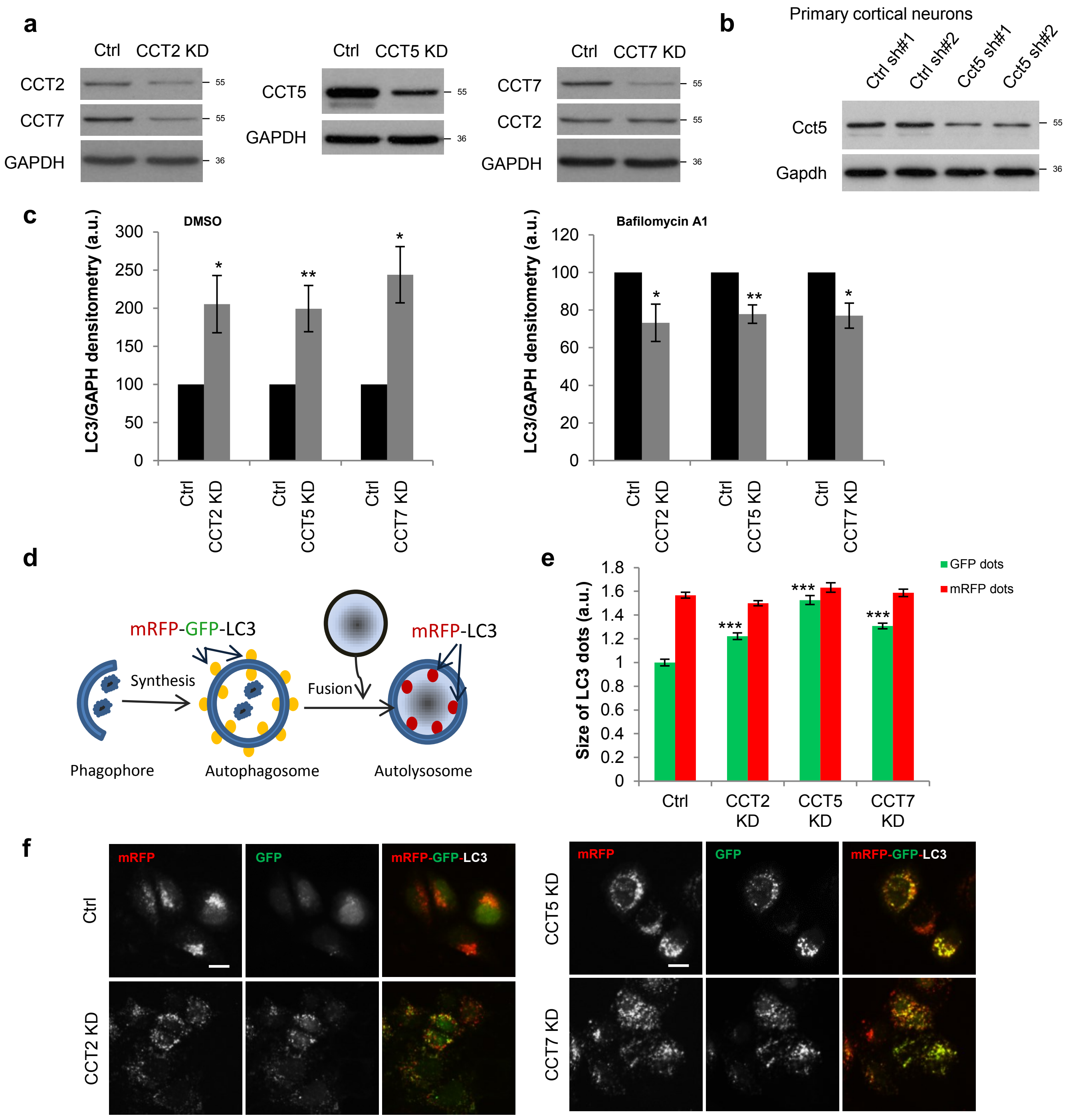

Supplementary Fig. 1. CCT knockdown efficiency.

(a) CCT depletion efficiency in HeLa cells. HeLa cells treated with siRNAs targeting individual CCT subunits were blotted for various subunits of the complex. Knockdown of one subunit can also impact on the levels of the others. GAPDH was used as loading control.

(b) CCT depletion efficiency in mouse primary cortical neurons. Primary cortical neurons were transduced with two different shRNAs targeting control (Ctrl sh#1 and sh#2) or the mouse Cct5 subunit (Cct5 sh#1 and sh#2). Gapdh was used as loading control.

(c) LC3-II/GAPDH densitometry for HeLa cells, related to Fig. 1a. The graphs show the mean ± SEM of independent biological replicates in the absence (n=6) or presence (n=4) of Bafilomycin A1. (**P<0.01, *P<0.05; two-tailed t-test).

(d) Schematic model for HeLa cells stably expressing mRFP-GFP-LC3. In this cell line, due to the different pKa of the two tags (< 4.5 for mRFP and around 6 for GFP), the autophagosomes emit both red (mRFP) and green (GFP) fluorescence, while the autolysosomes lose the GFP signal due to its rapid quenching in the acidic lysosomal compartment and are visualized as red-only vesicles by microscopy (Kimura et al., 2007; Sarkar et al., 2011).

(e) Size of GFP dots (autophagosomes/non-acidified lysosomes) and mRFP only dots (autolysosomes). HeLa cells stably expressing mRFP-GFP-LC3 were treated as in Fig. 1e, then seeded in a 96 well plate and subjected to Cellomics visualization analysis. The graphs show the results for one representative experiment: 12 wells of 100-200 cells each were analysed per sample. Similar data was seen in other two independent experiments. Bars represent the means ± SEM (n=12; ***P<0.001; two-tailed t-test).

(f) Representative Cellomics fields for the experiment in **(e)**. Scale bar throughout the panel is 10 μm.

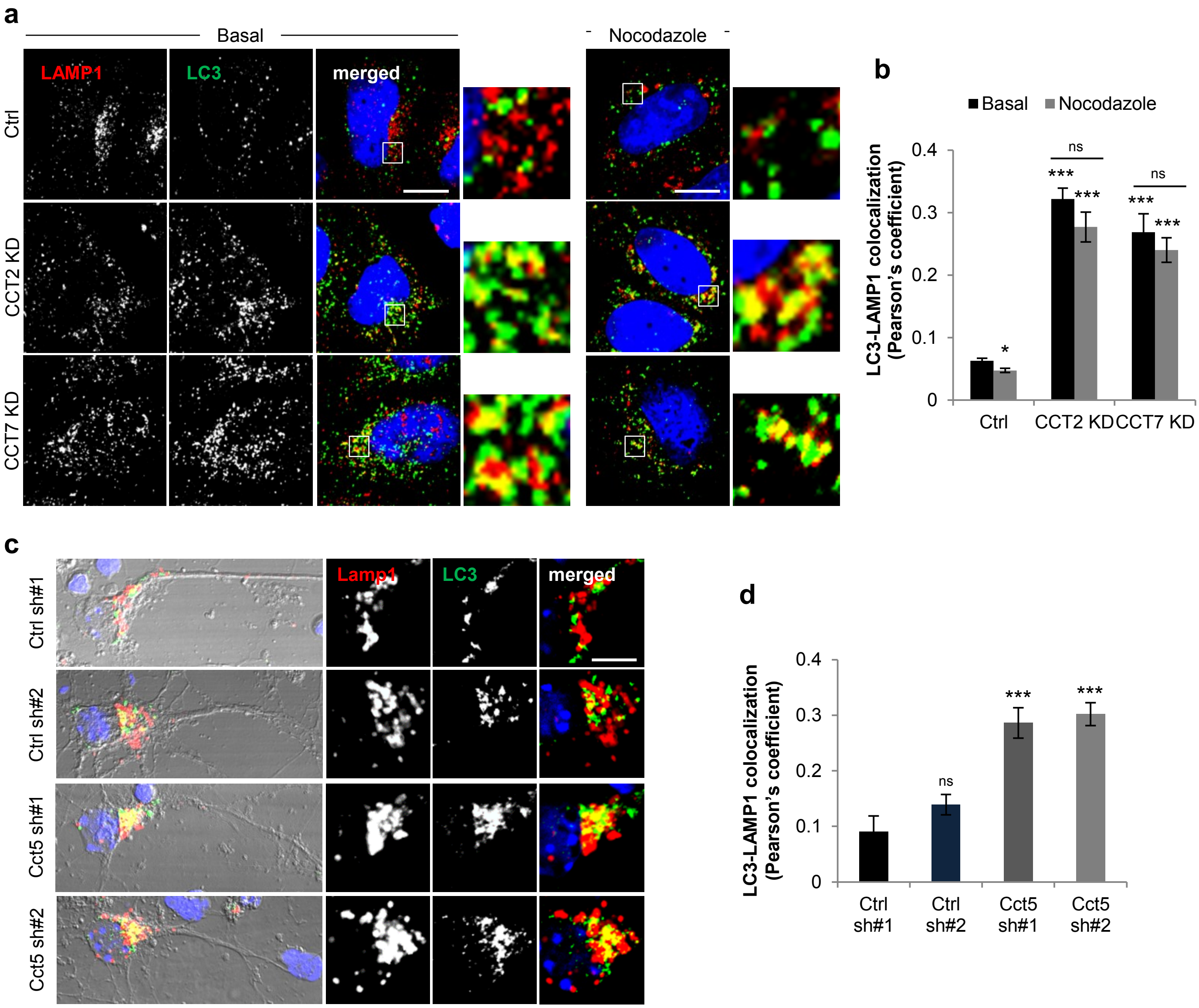

Supplementary Fig. 2. Loss of CCT function does not alter the autophagosome-lysosome fusion

(a) LC3 (anti-mouse Alexa 488) and LAMP1 (anti-rabbit Alexa 568) colocalization in HeLa cells. Cells depleted for CCT2 or CCT7 were co-imunostained for LAMP1 and LC3. The cells were then exposed to nocodazole treatment 20 µM, 3 hours. Scale bar throughout the panel is 10 µm.

(b) Quantification of colocalization for the experiment in **(a)**. The Pearson's coefficient was quantified for more than 30 cells per each condition for both basal and nocodazole treated cells. Bars represent the mean ± SEM (***P<0.001, *P<0.05, ns – not significant; two-tailed t-test). Similar results were achieved in at least two independent experiments.

(c) LC3 (anti-mouse Alexa 488) and LAMP1 (anti-rabbit Alexa 647) colocalization in mouse primary cortical neurons. The cortical neurons were transduced with two independent shRNAs targeting control or the mouse Cct5. Scale bar throughout the panel is 10 µm.

(d) Pearson's coefficients for the experiment in **(c)**. 20-35 neurons were considered per condition. Bars represent the mean ± SEM (***P<0.001, ns – not significant; two-tailed t-test). The experiment was repeated with similar results.

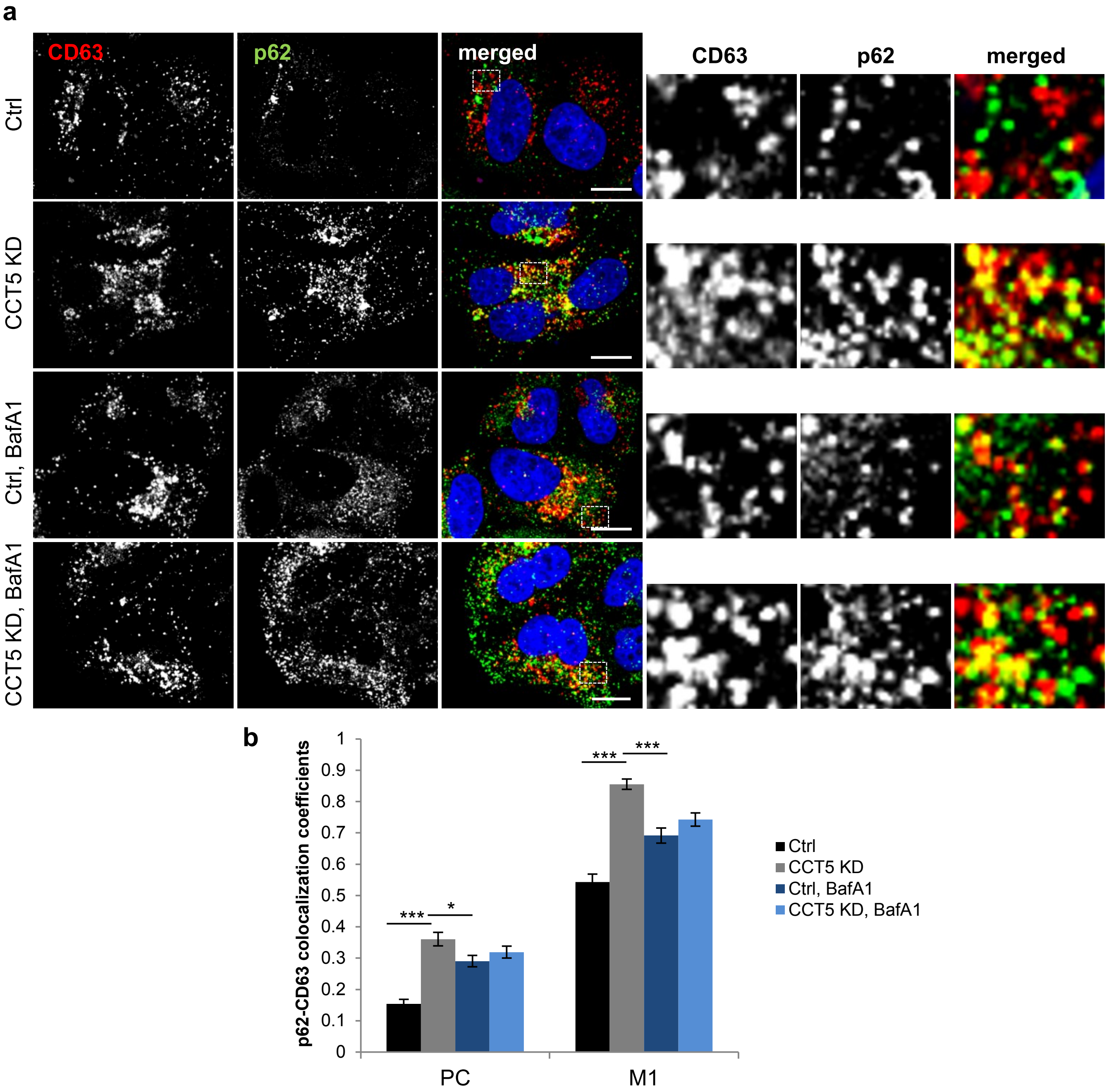

Supplementary Fig. 3. CCT depletion increases the p62 delivery into lysosomes.

(a) P62 (anti-rabbit Alexa 488) and CD63 (anti-mouse Alexa 568) colocalization in HeLa cells. Cells depleted for CCT5, in the absence or presence of Bafilomycin A1 (BafA1), were co-imunostained for CD63 and p62. Scale bar throughout the panel is 10 µm.

(b) Quantification of colocalization for the experiment in **(a)**. The Pearson's (PC) and Mander's (M1 represents the p62 localization in the lysosomal compartment) coefficients were quantified for more than 40 cells per each condition. Bars represent the mean ± SEM (***P<0.001, *P<0.05; two-tailed t-test). The experiment was repeated with similar results.

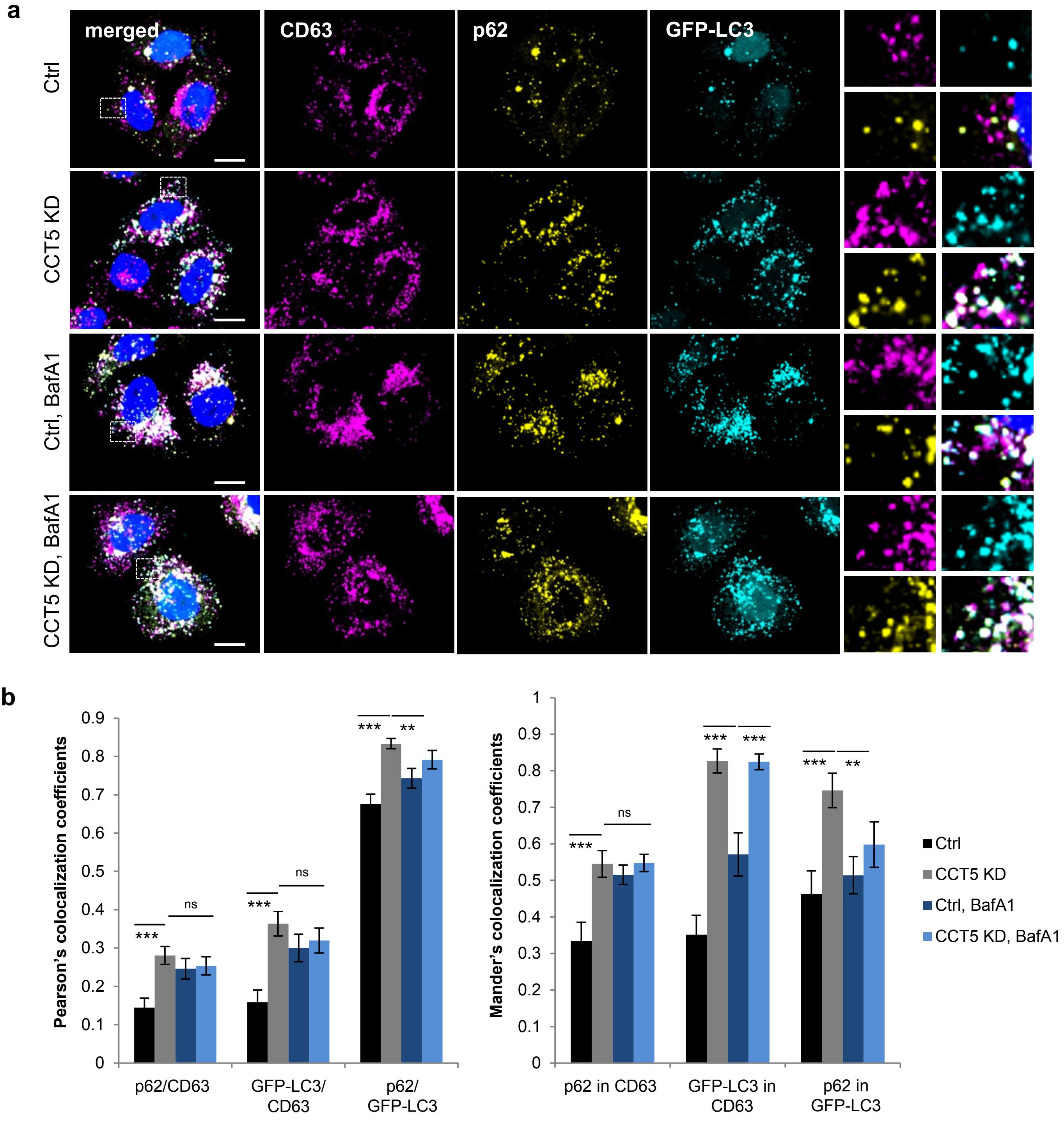

Supplementary Fig. 4. CCT depletion increases the p62 delivery into autophagosomes/autolysosomes.

(a) P62 (anti-rabbit Alexa 594) - CD63 (anti-mouse Alexa 647) – GFP-LC3 colocalization in HeLa cells stably expressing GFP-LC3. Cells depleted for CCT5, in the absence or presence of Bafilomycin A1 (BafA1), were co-imunostained for CD63 and p62. Scale bar throughout the panel is 10 µm.

(b) Quantification of colocalization for the experiment in **(a)**. The Pearson's and Mander's coefficients were quantified for more than 20 cells per each condition. Bars represent the mean ± SEM (***P<0.001, *P<0.05; two-tailed t-test). The experiment was repeated with similar results.

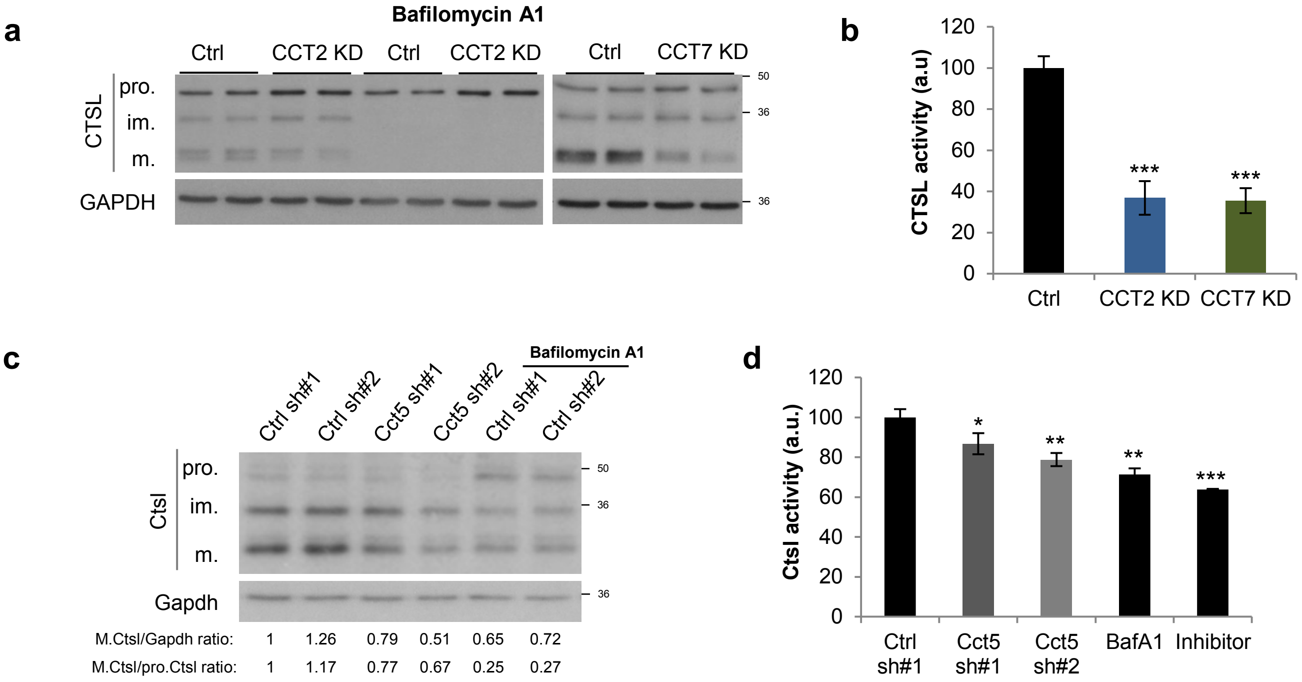

Supplementary Fig. 5. Loss of CCT function reduces cathepsin L maturation.

(a) Cathepsin L (CTSL) maturation immunoblot in CCT2 and 7 knockdown HeLa cells. GAPDH was used as loading control.

(b) In vitro cathepsin L activity. HeLa cells depleted for CCT2 and 7 were subjected to the cathepsin L activity kit and the fluorescence intensity was expressed as percentage of control. Bars represent the mean ± SD for one representative experiment (n=3; ***$P<0.001$; two-tailed t-test).

(c) Cathepsin L (Ctsl) maturation immunoblot in Cct5 depleted primary mouse cortical neurons. Gapdh was used as loading control.

(d) In vitro cathepsin L activity in primary cortical neurons exposed to shRNA lentiviral treatment as indicated. Bars represent the mean ± SD (n=3; ***$P<0.001$, **$P<0.01$, *$P<0.05$; two-tailed t-test).

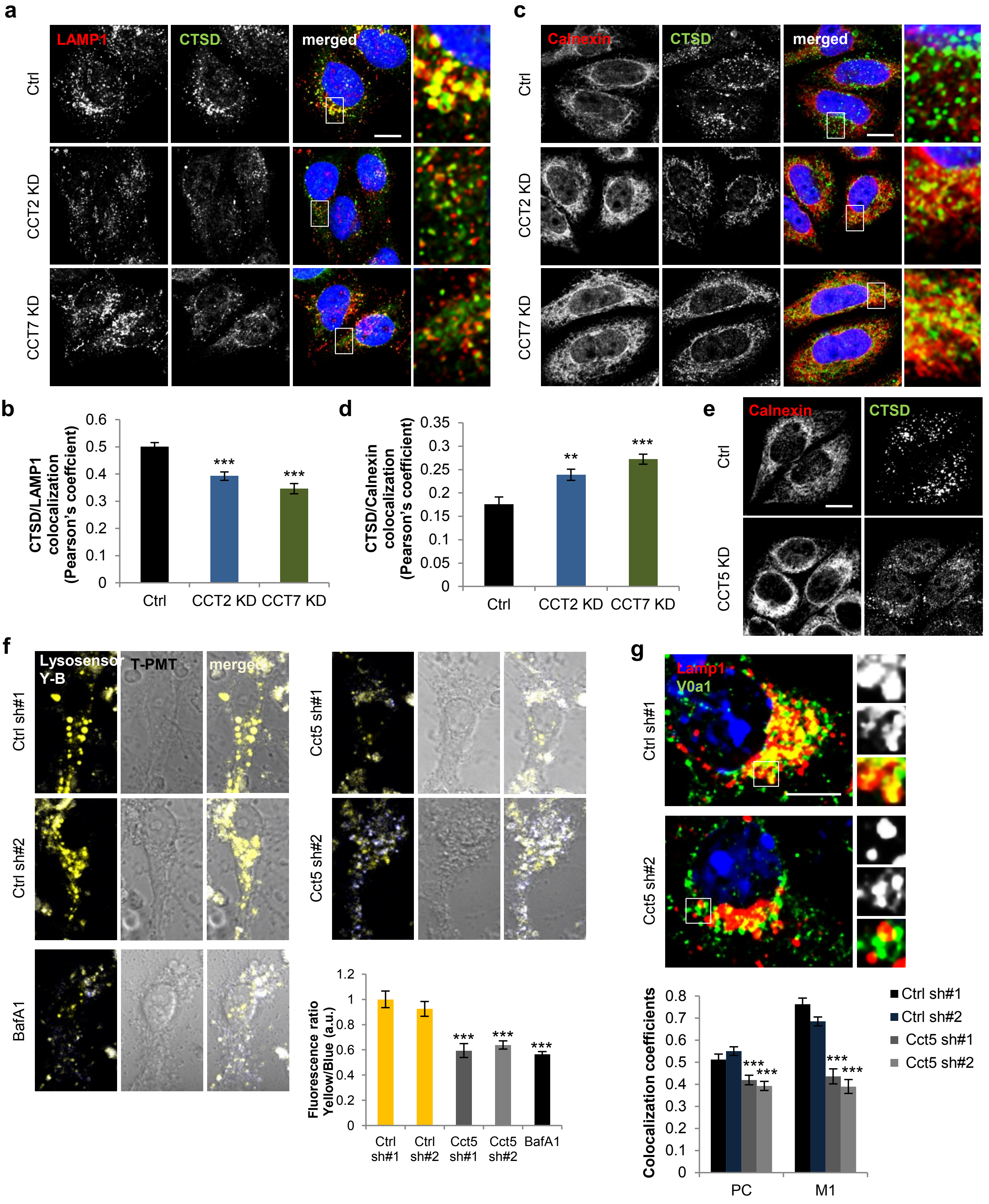

Supplementary Fig. 6. Cathepsin L maturation and lysosomal pH defects in CCT depleted cells.

(a) Cathepsin D (CTSD, anti-mouse Alexa 488) - LAMP1 (anti-rabbit Alexa 568) double immunostaining in CCT2 or CCT7 knockdown HeLa cells. Scale bar throughout the panel is 10 μm.

(b) CTSD-LAMP1 colocalization for the experiment in **(a)**. At least 40 cells were quantified per experiment (each experiment was performed at least twice with similar results). Bars represent the mean ± SEM (***P<0.001; two-tailed t-test).

(c) Cathepsin D (anti-mouse Alexa 488) – Calnexin (anti-rabbit Alexa 568) double immunostaining in CCT2 or CCT7 knockdown HeLa cells. Scale bar throughout the panel is 10 µm.

(d) CTSD-Calnexin colocalization for the experiment in **(c)**. At least 40 cells were quantified per experiment (each experiment was performed at least twice with similar results). Bars represent the mean ± SEM (***P<0.001, **P<0.01; two-tailed t-test).

(e) Enlarged image of cathepsin D (anti-mouse Alexa 488) – calnexin (anti-rabbit Alexa 568) double immunostaining from Fig. 2d. Scale bar throughout the panel is 10 µm.

(f) Representative images for the Lysosomal pH assay performed in Cct5-depleted primary cortical neurons using the Lysosensor Yellow-Blue. T-PMT states for the Bright Field/DIC image. Scale bar throughout the panel is 10 µm. See Fig. 2f.

(g) Voa1 (anti-rabbit Alexa555) and Lamp1 (anti-rat Alexa647) double immunostaining in mouse primary cortical neurons. Neurons were plated and treated as in Fig. 2g. At least 30 neurons were counted per condition. PC states for the Pearson's coefficient, while M1 is the Mander's coefficient 1, which indicates the Voa1 localization in the Lamp1 compartment. Bars represent the mean ± SEM (***P<0.001; two-tailed t-test). Scale bar throughout the panel is 10 µm.

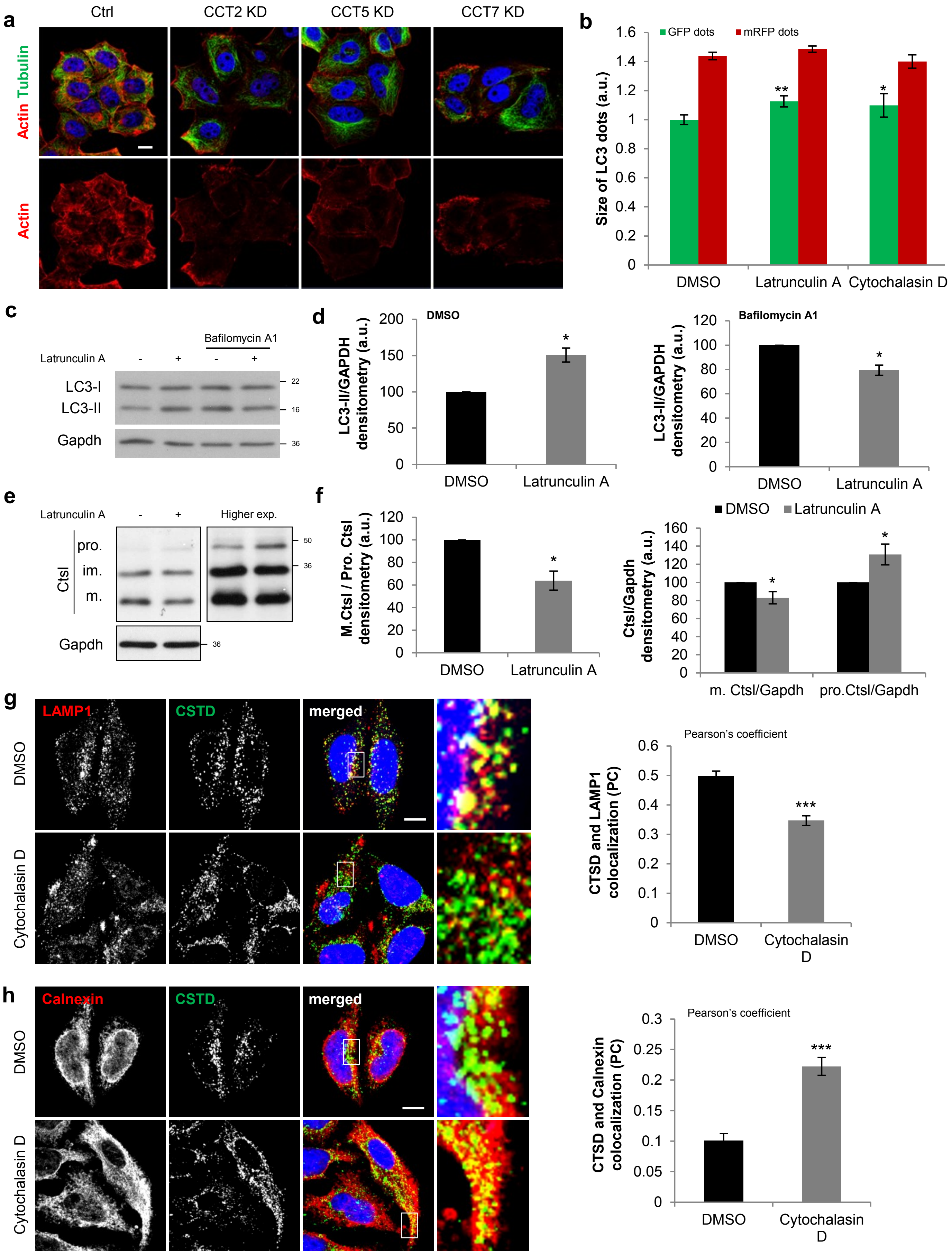

Supplementary Fig. 7. Actin depolymerising drugs blocks autophagy.

Supplementary Fig. 7. Actin depolymerising drugs blocks autophagy.

(a) Cytoskeleton immunostaining in CCT knockdown cells. Scale bar throughout the panel is 10 µm.

(b) Size of GFP dots (autophagosomes/non-acidified lysosomes) and mRFP only dots (autolysosomes) in mRFP-GFP-LC3 stable HeLa cells independently treated with the actin depolymerising drugs - latrunculin A and cytochalasin D for 3 hours at 1 µM concentration each, and subjected to Cellomics visualization analysis. The graphs show the results for one representative experiment: 8 wells of 100-200 cells each were analysed per sample. The experiment was repeated with similar results. Bars represent the means ± SEM (n=8; $^{**}P<0.01$, $^{*}P<0.05$; two-tailed t-test).

(c) LC3-II levels in mouse primary cortical neurons treated with latrunculin A. Primary cortical neurons (DIV 7) were exposed to Latrunculin A (1 µM) for 6 hours in the presence or absence of Bafilomycin A1 (400 nM, 6 hours).

(d) LC3-II/Gapdh densitometry for the experiment in **(c)**. Bars represent the mean ± SD (n=3; $^{*}P<0.05$; two-tailed one sample t-test).

(e) Cathepsin L (Ctsl) maturation immunoblot in primary neurons treated with Latrunculin A (1 µM) for 6 hours (pro = pro-Cathepsin L, im = intermediate Cathepsin L, m = mature Cathepsin L).

(f) Quantification of m.Ctsl/pro.Ctsl, m.Ctsl/Gapdh and pro.Ctsl/Gapdh ratios in primary cortical neurons treated as in **(e)**. For the first two, the statistical analysis was based on two-tailed one sample t-test and for the pro.Ctsl/Gapdh ratio, one-tailed one sample t-test (n=3; $^{*}P<0.05$).

(g) Cathepsin D (CTSD, anti-mouse Alexa 488) and LAMP1 (anti-rabbit Alexa 568) double immunostaining in 1 µM cytochalasin D-treated cells for 3 hours. The quantification of colocalization is displayed on the right panel. At least 30 cells were counted per experiment (each experiment was performed at least twice). Bars represent the mean ± SEM ($^{***}P<0.001$; two-tailed t-test). Scale bar throughout the panel is 10 µm.

(h) Cathepsin D (anti-mouse Alexa 488) and calnexin (anti-rabbit Alexa 568) double immunostaining in 1 µM cytochalasin D treated cells for 3 hours. The quantification of colocalization is displayed on the right panel. At least 30 cells were counted per experiment (each experiment was performed at least twice). Bars represent the mean ± SEM ($^{***}P<0.001$; two-tailed t-test). Scale bar throughout the panel is 10 µm.

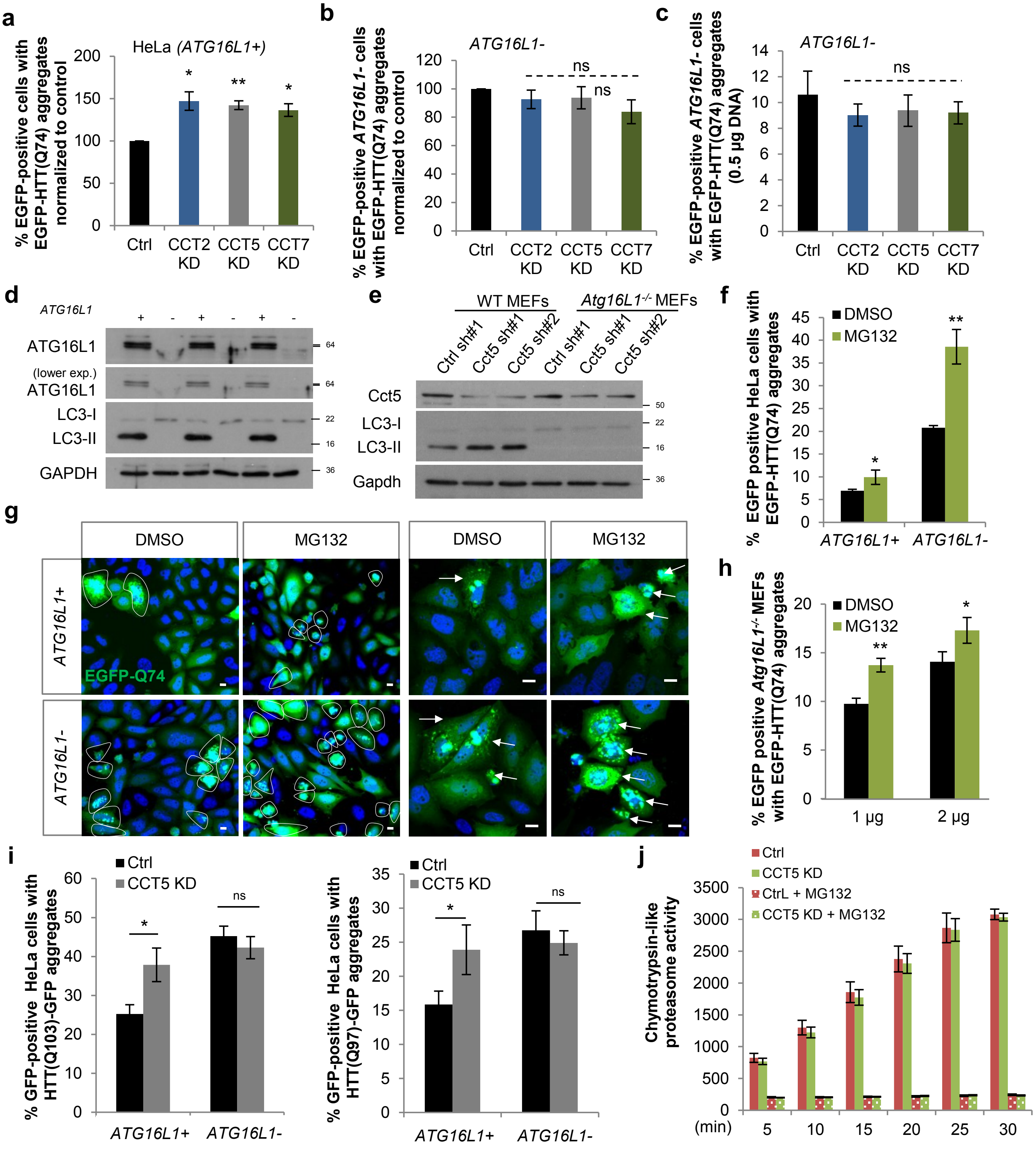

Supplementary Fig. 8. CCT knockdown monitors the EGFP-HTT(Q74) aggregation through autophagy.

(a) EGFP-HTT(Q74) aggregation in HeLa (*ATG16L1+*) cells exposed to siRNA targeting different CCT subunits. The bars represent the mean of data from 3 independent experiments (biological replicates) ± SD (n=3; **P<0.01, *P<0.05; one sample t-test), while each experiment was performed in triplicates.

(b) EGFP-HTT(Q74) aggregation in (*ATG16L1-)* CRISPR/Cas9 HeLa cells exposed to siRNA targeting different CCT subunits. The bars represent the mean of data from 3 independent experiments ± SD (n=3; ns – not significant; one sample t-test), while each experiment was performed in triplicates.

(c) EGFP-HTT(Q74) aggregation in (*ATG16L1-)* CRISPR/Cas9 HeLa cells exposed to siRNA targeting different CCT subunits. The experiment was performed in parallel with the one shown in Fig. 5b. The cells were seeded on coverslips in triplicates, transfected with siRNAs targeting control or individual CCT subunits and followed by 0.5 µg of EGFP-HTT(Q74). The bars represent the mean of the percentages of cells with aggregates ± SD (n=3; ns – not significant; two-tailed t-test).

(d) Biochemical assessment of *ATG16L1-* HeLa. CRISPR/Cas9 mediated degradation of *ATG16L1* in HeLa cells shows no residual autophagy or ATG16L1. GAPDH was used as loading control.

(e) Validation of Cct5 depletion in WT and *Atg16L1*$^{-/-}$ MEFs. The MEFs were transduced with lentiviral particles targeting control or the mouse Cct5 subunit (two independent shRNAs). LC3-II and Cct5 levels were assessed by western blot. Gapdh was used as loading control.

(f) *ATG16L1+* and *ATG16L1-* HeLa cells were seeded on coverslips in triplicates and transfected with 1 µg of EGFP-HTT(Q74) and treated with vehicle (DMSO) or MG132 (10 µM) for the last 12 hours. The bars represent the mean of the percentages of cells with aggregates ± SD (n=3; **P<0.01, *P<0.05; two-tailed t-test). Treatment with MG132 further increased the percentage of *ATG16L1-* HeLa cells with EGFP-HTT(Q74) aggregates.

(g) Representative images for DMSO and MG132 treated cells as in **(f)**. Scale bar throughout the panel is 10 µm. The cells with aggregates are indicated with white lines or arrows.

(h) *Atg16L1-/-* MEFs were seeded on coverslips in triplicates and transfected with 1 µg or 2 µg of EGFP-HTT(Q74) and treated with vehicle (DMSO) or MG132 (10 µM) for the last 12 hours. Left: The bars represent the mean of the percentages of cells with aggregates ± SD (n=3; **P<0.01, *P<0.05; two-tailed t-test). Treatment with MG132 increased the percentage of Atg16L1-/- MEFs with Q74-GFP aggregates.

(i) N17-103Q-GFP (left) and N17-97QP-GFP (right) aggregation in CCT-depleted *ATG16L1+* and *ATG16L1-* HeLa cells from one representative experiment performed in triplicates. The bars represent the mean of the percentages of cells with aggregates ± SD (n=3; *P<0.05, ns – not significant; one sample t-test). Similar results were achieved in other three independent experiments.

(j) Chymotrypsin-like proteasome activity in CCT5 knockdown HeLa cells (n=3; ns – not significant; two-tailed t-test).

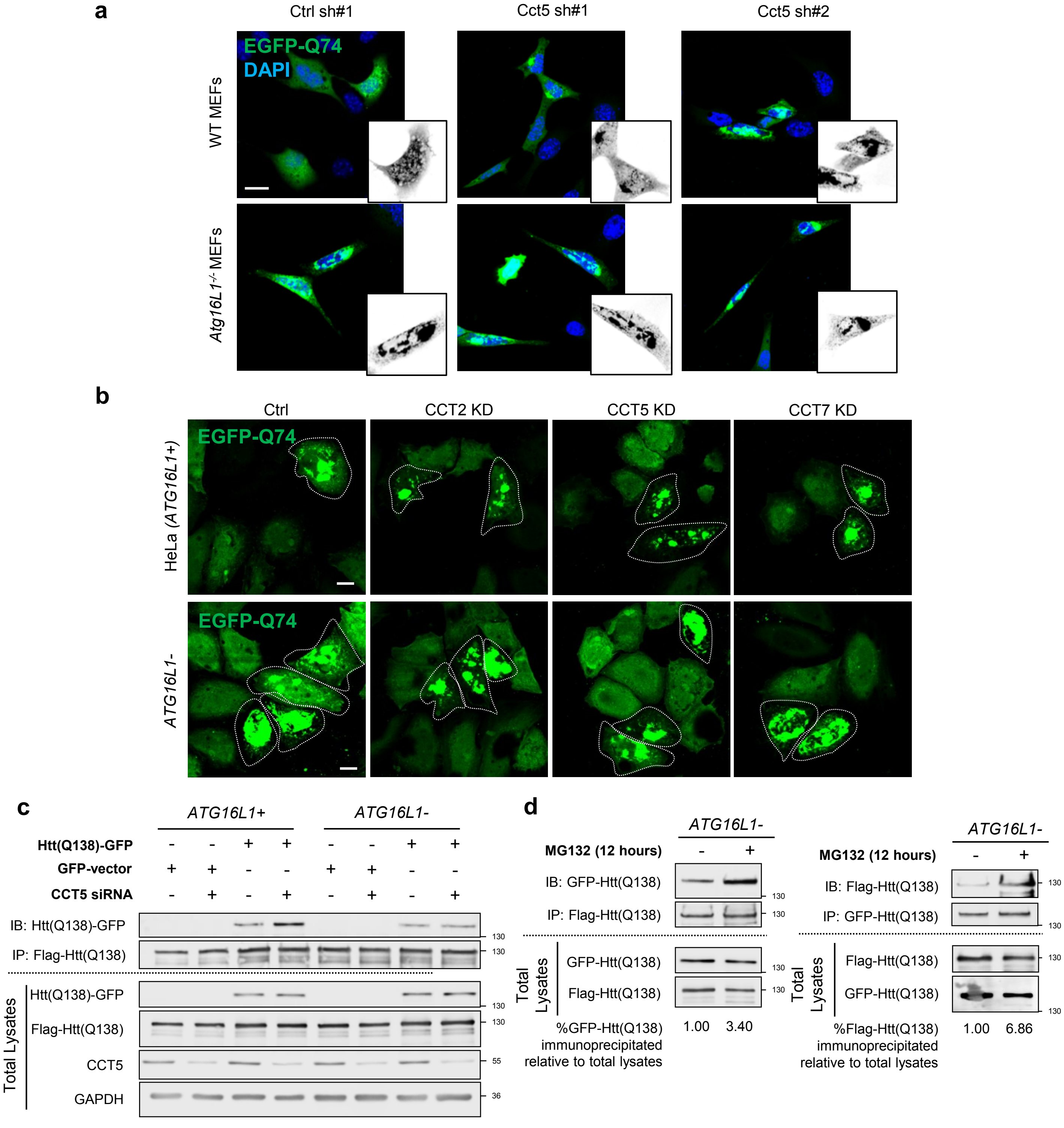

Supplementary Fig. 9. EGFP-HTT(Q74) aggregates in CCT knockdown cells

(a) Representative images of WT and *Atg16L1*$^{-/-}$ MEFs with EGFP-HTT(Q74) aggregates for the experiment presented in Fig. 5c. Cells were seeded on coverslips in triplicates, transduced with lentiviral particles targeting control or the mouse Cct5 subunit (two independent shRNAs) and transfected with EGFP-HTT(Q74). Similar data was achieved in other two independent experiments.

(b) Upper: Representative images of HeLa (*ATG16L1+*) cells with Q74-GFP aggregates treated with siRNA targeting various CCT subunits, as indicated in Fig. 5a.

Bottom: Representative images of (*ATG16L1-)* CRISPR/Cas9 HeLa cells with EGFP-HTT(Q74) aggregates treated with siRNA targeting various CCT subunits, as indicated in Fig. bB. The dotted lines indicate the cells with EGFP-HTT(Q74) aggregates. Scale bar throughout the panel is 10 µm.

(c) Full western-blot for Fig. 6a.

(d) Oligomerization of mutant htt in *ATG16L1-* HeLa cells exposed to either vehicle (DMSO) or MG132 (10 µM) for the last 12 hours (see Methods and Fig. 6a-c). Treatment with MG132 enhanced the amount of coimunoprecipitated of mutant HTT(1-548) tagged to GFP with Flag-tagged mutant HTT(1-588) – see left panel, and *vice-versa* – see right panel*,* in the autophagy null cells.

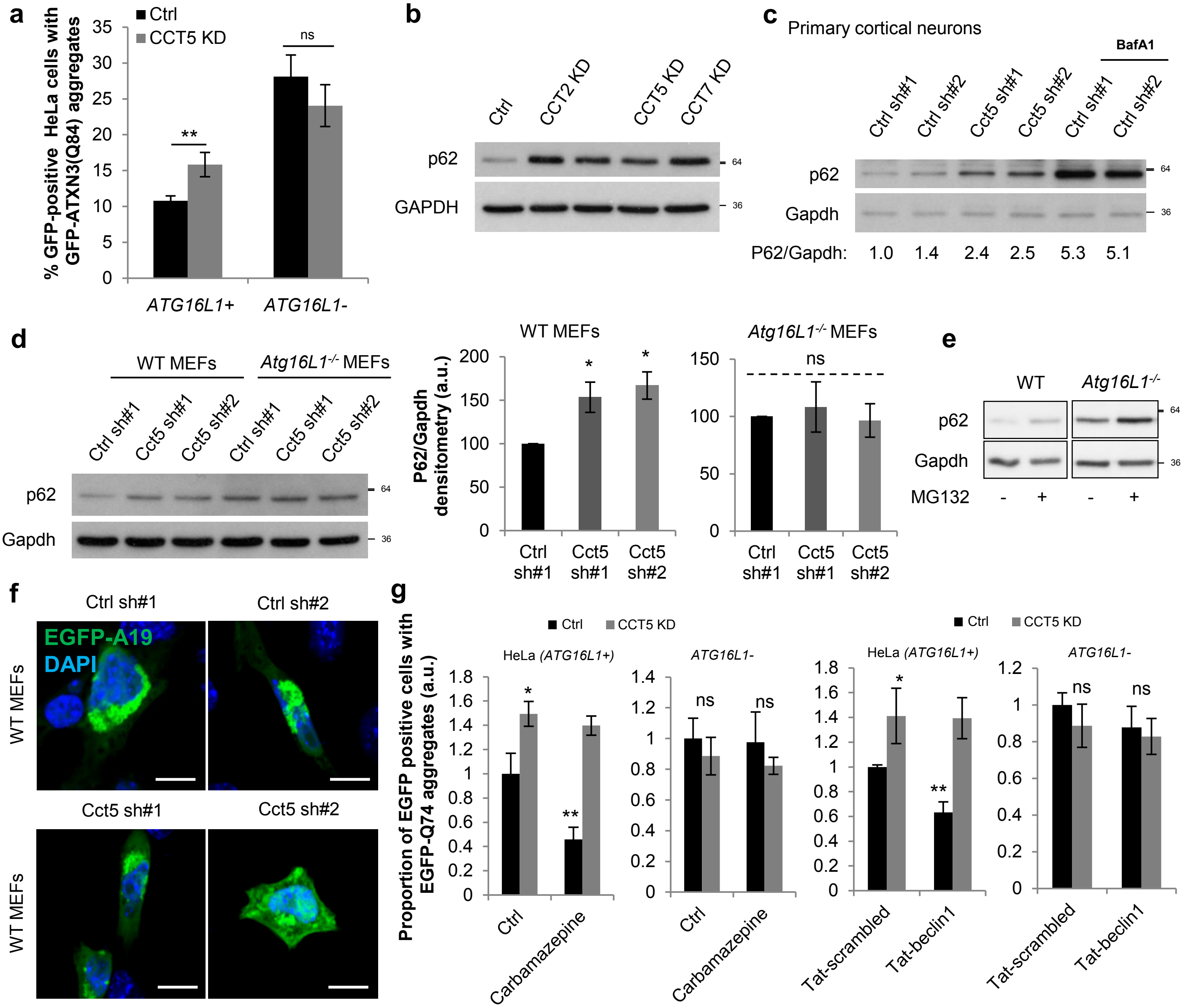

Supplementary Fig. 10. CCT depletion increases the accumulation of autophagy substrates

(a) GFP-ATXN3(Q84) aggregation in *ATG16L1+* and *ATG16L1-* HeLa cells. Cells seeded on coverslips in triplicates, were exposed to either control or CCT5 siRNA and transfected with GFP-ATXN3(Q84). The bars represent the mean of percentages of cells with aggregates ± SD (n=3; **P<0.01, ns – not significant; two-tailed t-test). Similar results were achieved in other three independent experiments.

(b) P62 levels in HeLa cells transiently transfected with siRNA targeting individual CCT subunits.

(c) P62 levels in mouse primary cortical neurons. Primary cortical neurons were transduced with lentiviral particles targeting Cct5. Two independent shRNAs were used per each condition. Bafilomycin A1 (400nM for 12 hours) was used as a positive control for the accumulation of the autophagy substrates, p62. P62/Gapdh densitometry is shown bellow.

(d) P62 levels in WT and *Atg16L1-/-* MEFs transduced with shRNA lentiviral particles targeting control or the mouse Cct5 subunit. The bars represent the mean of three independent experiments ± SD (n=3; *P<0.05, ns – not significant; two-tailed one sample t-test).

(e) P62 levels in WT and *Atg16L1*$^{-/-}$ MEFs treated with vehicle (DMSO) or MG132 (10 µM) for 4 hours. MG132 increases the p62 levels in both WT and *Atg16L1*$^{-/-}$ MEFs.

(f) Representative images of EGFP-A19 aggregates in WT MEFs transduced with shRNA lentiviral particles targeting control or the mouse Cct5 subunit. Similar data was achieved in other two independent experiments.

(g) EGFP-HTT(Q74) aggregation in *ATG16L1+* and *ATG16L1-* HeLa cells exposed to either control or CCT5 siRNA and treated with the autophagy inducers: carbamazepine (50 µM) and Tat-Beclin1 peptide (20 µM). HeLa cells were exposed to two round of siRNA, transfected with EGFP-HTT(Q74) and treated with autophagy inducers for the last 36 hours. The percentage of transfected cells with aggregates was scored and the control data were normalised to 1 to enable comparisons. Note that the effects in CCT-depleted cells were observed in two independent triplicate experiments. Data are shown for representative triplicate experiments with t-tests.

a

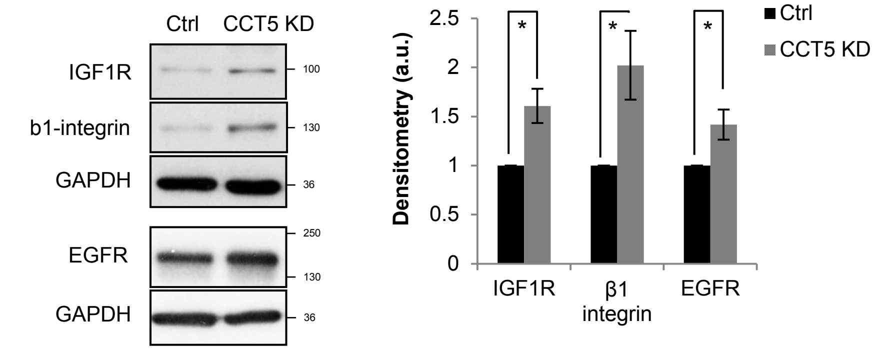

b

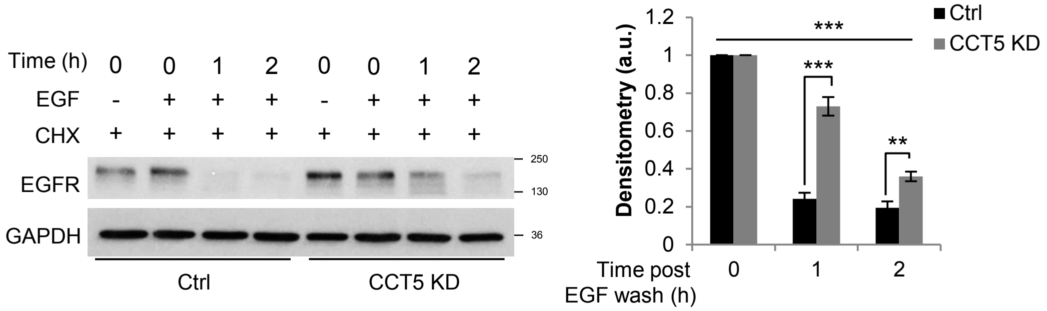

Supplementary Fig. 11. Accumulation of endocytic substrates upon CCT5 depletion

(a) Protein levels of known endocytic route cargos in CCT5 siRNA knockdown HeLa cells. CCT5 KD significantly increased the levels of IGF1R, β1-integrin and EGFR. The bars represent the mean of data ± SD (n=3; *P<0.05; two-tailed one sample t-test).

(b) EGFR degradation assay in HeLa cells exposed to either Ctrl or CCT5 siRNA. HeLa cells were serum starved (DMEM with 0.2% FBS) for 4 hours before loading with the receptor ligand EGF (100 ng/ml for 30 min) in the presence of cyclohexamide (CHX, 40 µg/ml). After, the cells were washed twice with PBS, and further incubated in serum-free media in the presence of cyclohexamide for another 1 or 2 hours. At "time 0" the cells were lysed immediately after the PBS wash. The graph represents the quantification of EGFR degradation in HeLa cells exposed to either Ctrl or CCT5 siRNA. For each individual time point we normalized the data to "time 0" (EGF/CHX) and used two-tailed t-test to assess the protein level differences between Ctrl and CCT5 depleted cells. ANOVA two ways was performed to assess the overall significance of EGFR degradation. The bars represent the mean of data ± SD (n=3; ***P<0.001, **P<0.01; two tailed one sample t-test). CCT5 KD reduces the EGFR degradation. See Materials and Methods.

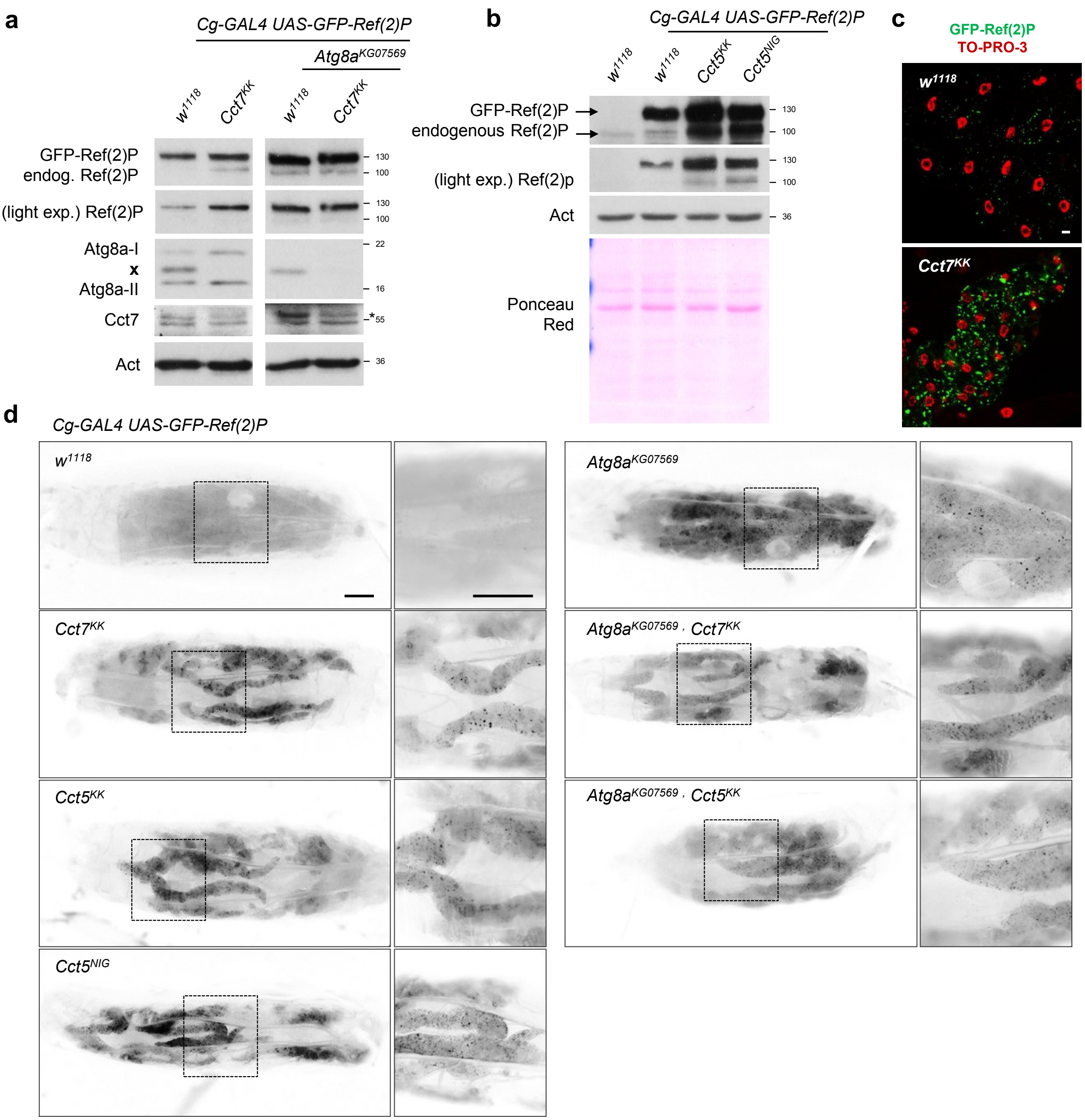

Supplementary Fig. 12. CCT depletion blocks autophagy in *Drosophila*.

(a) Biochemical assessment of autophagy in the fat body of *Cg-GAL4 UAS-GFP-Ref(2)P* third instar larvae upon *Cct7* knockdown, either alone (*Cct7*^{KK}) or in an *Atg8a* mutant background (*Atg8a*^{KG07569}). The VDRC $w^{111\ 8}$ line (stock number 60100) was used as background control. Ref(2)P (both GFP-tagged and endogenous forms) accumulated upon *Cct7* knockdown (*Cct7*^{KK}) and in *Atg8a* mutants. Atg8-II levels increase in *Cct7*-depleted fat bodies. There was no obvious effect of *Cct7* knockdown on Ref(2)P levels in *Atg8a* mutant fat bodies. Actin was used as loading control.

(b) Biochemical assessment of endogenous and GFP-tagged Ref(2)P upon *Cct5* knockdown in the fat body of third instar larvae expressing GFP-Ref(2)P (*Cg-GAL4::UAS-GFP-Ref(2)P*) and *Cct5* RNAi (*Cct5*^{KK} or *Cct5*^{NIG}). The VDRC w^{1118} (stock number 60100) was used as a background control.

The western blot analysis was performed using a 10 % polyacrylamide gel, to better separate the GFP-tagged from the endogenous Ref2(P). Ponceau-Red staining validates actin as a loading control in *Drosophila* fat bodies.

(c) Representative confocal images showing GFP-Ref(2)P in the fat body larval progeny of *Cg-GAL4 UAS-GFP-Ref2(P)* crossed to either w^{1118} or $Cct7^{KK}$. GFP-Ref2(P) positive aggregates accumulates in *Cct7*-depleted cells. Scale bar throughout the panel is 10 µm.

(d) Macroscopic images showing GFP-Ref2(P) aggregates in *Cg-GAL4::UAS-GFP-Ref(2)P* and $Atg8a^{KG075569}$*;Cg-GAL4::UAS- GFP-Ref(2)P* larvae depleted for *Cct7* ($Cct7^{KK}$) or *Cct5* (either $Cct5^{KK}$ or $Cct5^{NIG}$). Scale bar throughout the panel is 200 µm.

Supplementary Fig. 13

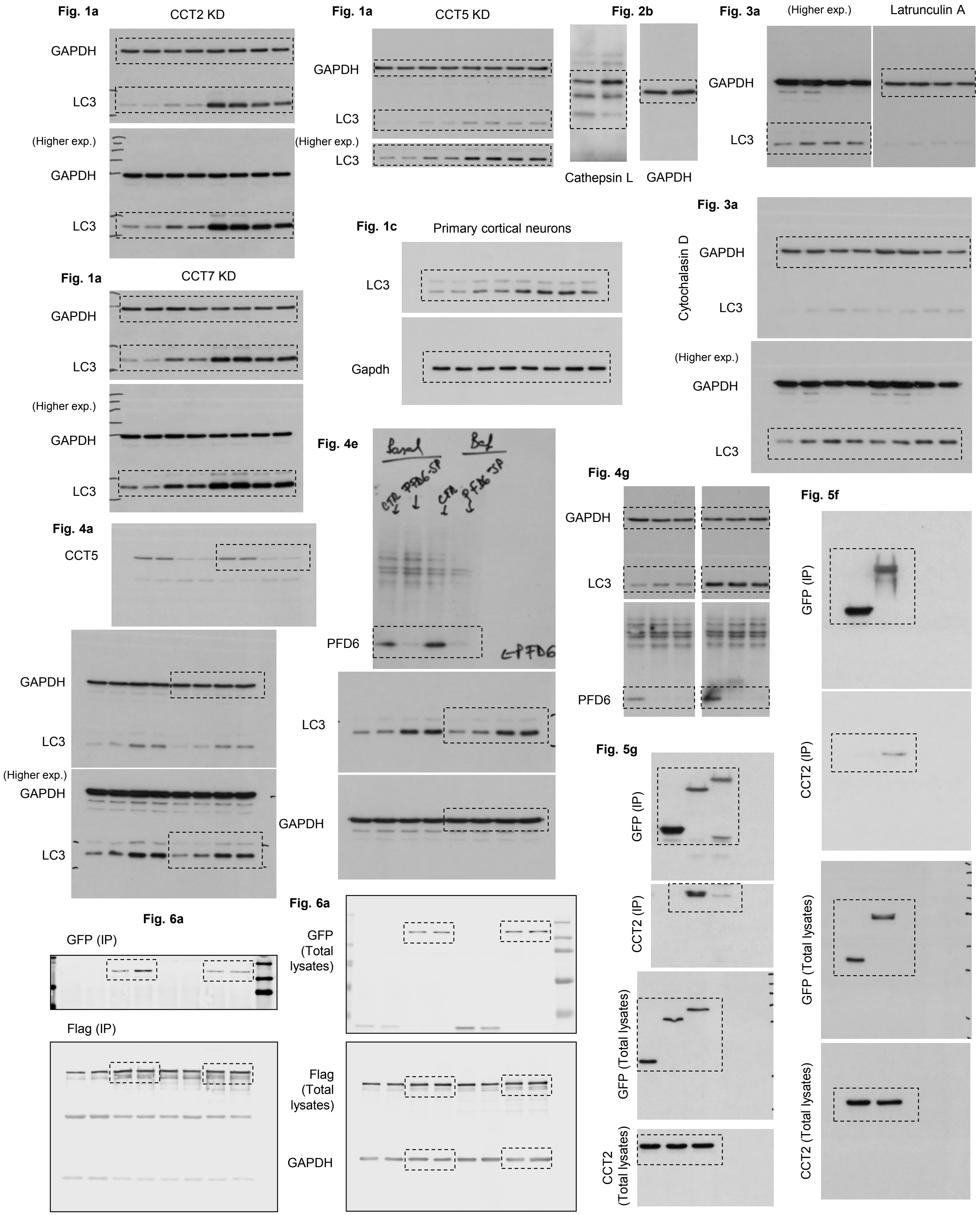

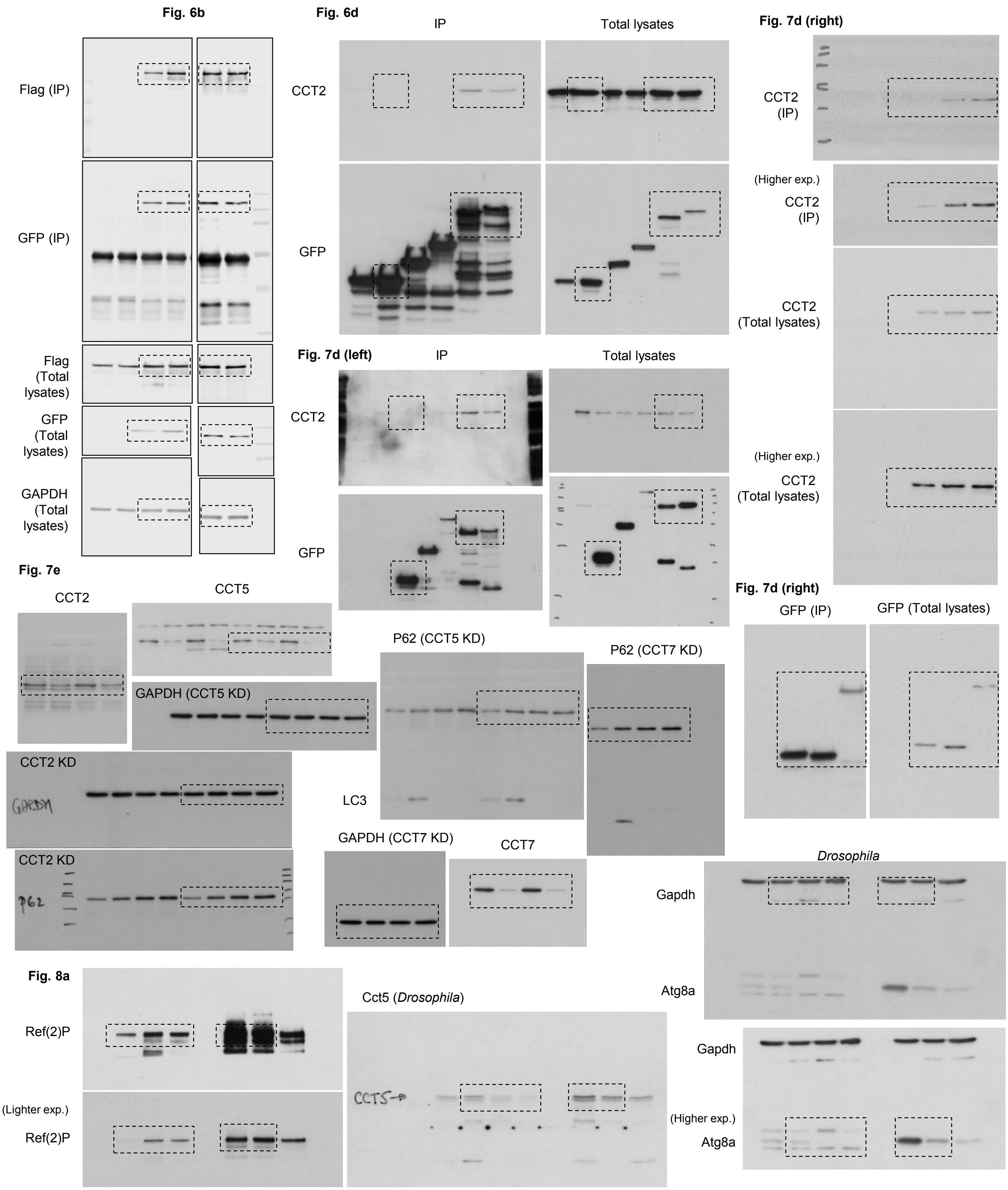
Fig. 6b
Flag (IP)
GFP (IP)
Flag (Total lysates)
GFP (Total lysates)
GAPDH (Total lysates)
Fig. 6d
IP
Total lysates
CCT2
GFP
Fig. 7d (left)
IP
Total lysates
CCT2
GFP
Fig. 7d (right)
CCT2 (IP)
(Higher exp.)
CCT2 (IP)
CCT2 (Total lysates)
(Higher exp.)
CCT2 (Total lysates)
Fig. 7e
CCT2
CCT5
GAPDH (CCT5 KD)
CCT2 KD
GAPDH
CCT2 KD
P62
P62 (CCT5 KD)
LC3
P62 (CCT7 KD)
GAPDH (CCT7 KD)
CCT7
Fig. 7d (right)
GFP (IP)
GFP (Total lysates)
Drosophila
Gapdh
Atg8a
Fig. 8a
Ref(2)P
(Lighter exp.)
Ref(2)P
Cct5 (*Drosophila*)
CCT5
Gapdh
(Higher exp.)
Atg8a

Supplementary Fig. 13 - continued

Supplementary Fig. 1a

CCT7 KD

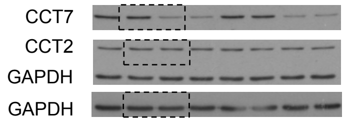

CCT2 KD

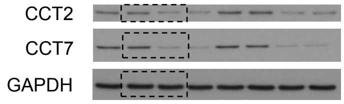

Supplementary Fig. 1a

Primary cortical neurons

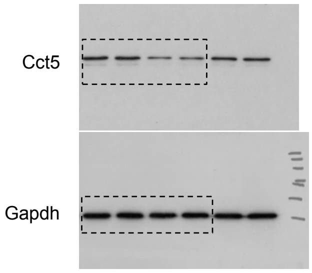

Supplementary Fig. 5a

Cathepsin L – CCT2 KD

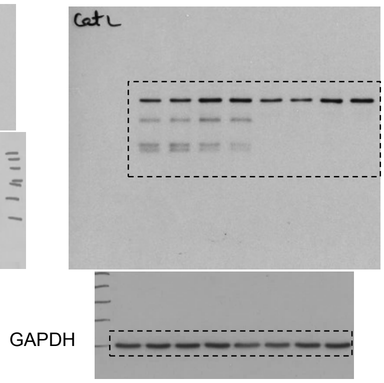

Cathepsin L – CCT7 KD

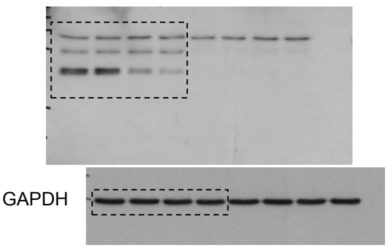

Supplementary Fig. 8d

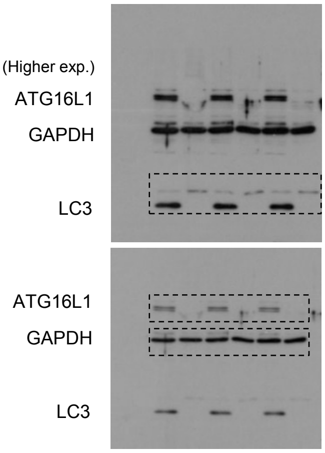

Supplementary Fig. 5c

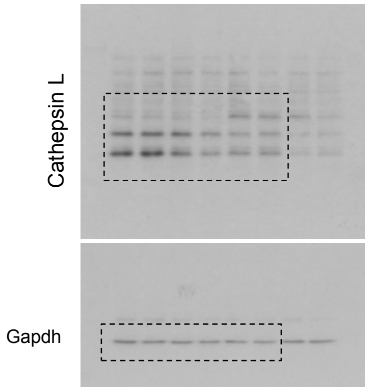

Supplementary Fig. 7c

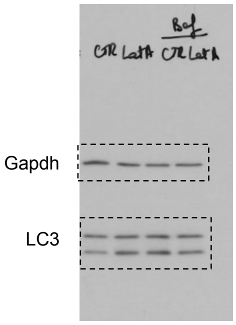

Supplementary Fig. 7e

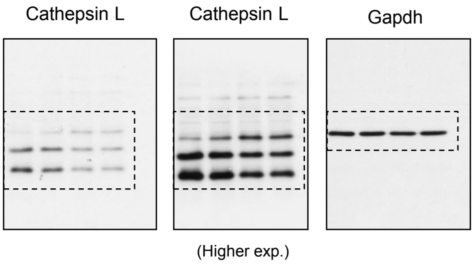

Supplementary Fig. 8e

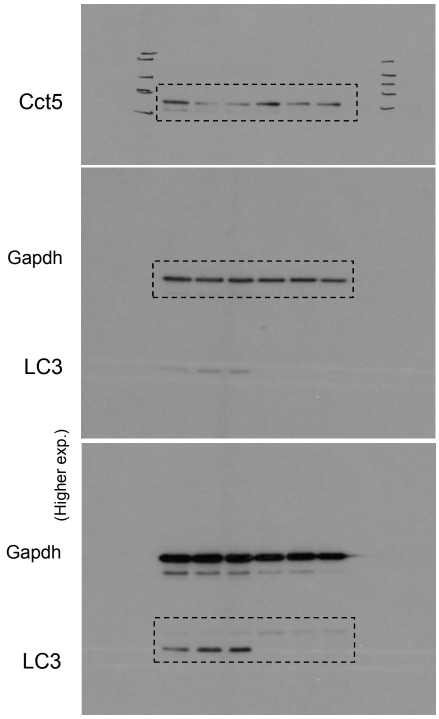

Supplementary Fig. 9d

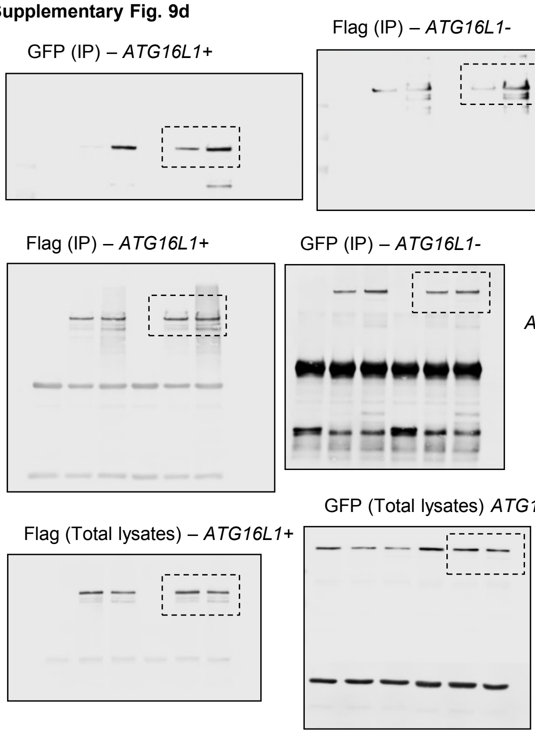

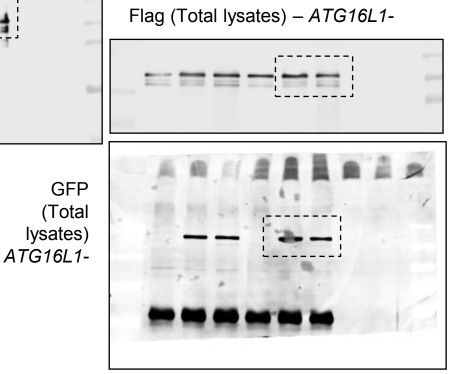

Supplementary Fig. 10b

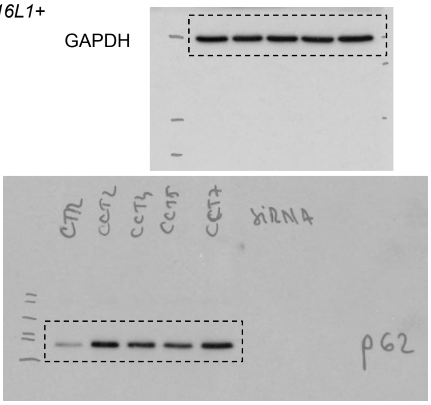

Supplementary Fig. 10c

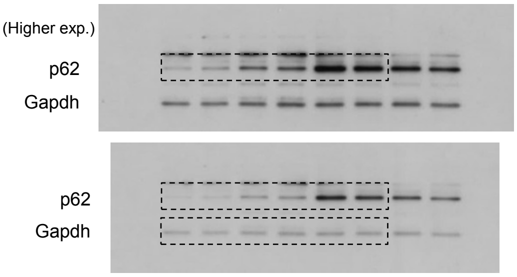

Supplementary Fig. 10d

Gapdh - MEFs

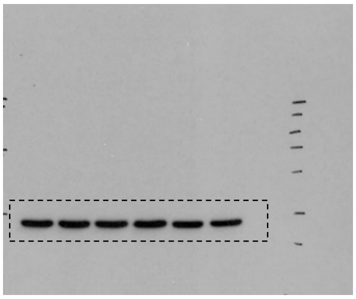

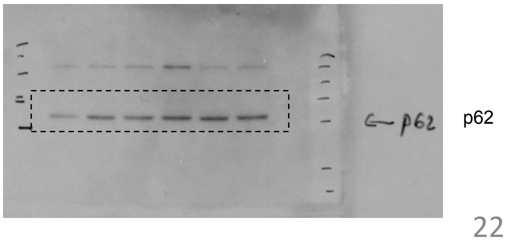

Supplementary Fig. 13 - continued

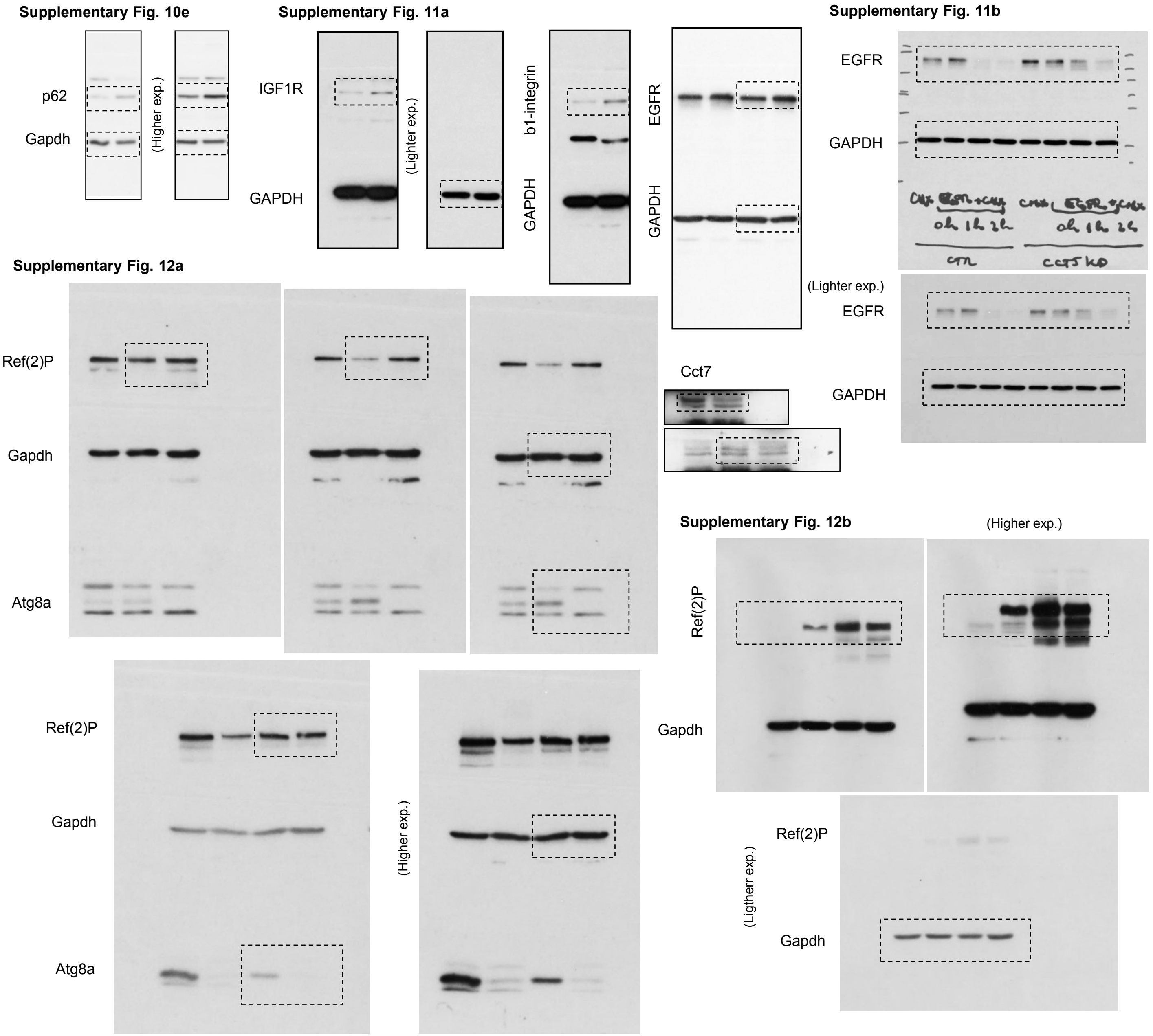

Supplementary Fig. 13. Full scans of uncropped blots.
